# Comparative Structure-Based Analysis of Predicted BZD9L1 Binding Modes Across Human Sirtuins

**DOI:** 10.3390/ijms27156840

**Published:** 2026-07-30

**Authors:** Yi Jer Tan, Yeuan Ting Lee, Chern Ein Oon, Ricardo L. Mancera

**Affiliations:** 1 Institute for Research in Molecular Medicine (INFORMM), Universiti Sains Malaysia, Penang 11800, Malaysia; richtan54@gmail.com (Y.J.T.); yeuanting93@gmail.com (Y.T.L.); 2 Curtin School of Diagnostic and Therapeutic Sciences, Curtin Medical Research Institute, Curtin University, GPO Box U1987, Perth, WA 6845, Australia

**Keywords:** BZD9L1, sirtuins, structure-based modelling, molecular docking, predicted binding modes, protein–ligand interactions

## Abstract

Sirtuins (SIRTs) are NAD^+^-dependent enzymes implicated in cancer and other diseases, but the high conservation of their catalytic sites complicates the development of isoform-selective inhibitors. BZD9L1 is a benzimidazole-based sirtuin inhibitor with previously reported activity against SIRT1 and SIRT2. However, its potential interactions with other human sirtuin isoforms remain incompletely characterized. Here, we applied a comparative structure-based modelling framework integrating homology modelling, molecular docking, and targeted experimental assessment to investigate plausible binding modes of BZD9L1 across human SIRT1–7. Docking predicted that BZD9L1 could occupy the ADP-ribose cofactor-binding region of all seven isoforms, with broadly conserved orientations but differences in the predicted interaction networks. Hydrogen-bonding and π-mediated contacts predominated in the selected SIRT1–3 poses, whereas hydrophobic contacts were more apparent in several selected SIRT4–7 poses. The modest differences in docking scores were interpreted qualitatively and do not establish differential binding affinities or isoform selectivity. In colorectal cancer cells, BZD9L1 treatment altered acetyl-SOD2 levels, consistent with altered SIRT3-associated deacetylation in a cellular context. In a separate cell-free enzymatic assay, no measurable SIRT5 inhibition was detected under the conditions tested. These complementary assays provided distinct, independently interpreted readouts of SIRT3-associated cellular activity and SIRT5 enzymatic activity. Collectively, this study provides testable structural hypotheses regarding BZD9L1 recognition by human sirtuins and identifies interaction features that may guide subsequent biochemical and structure-based investigations.

## 1. Introduction

Sirtuins (SIRTs) are NAD^+^-dependent enzymes that regulate diverse cellular processes, including aging, DNA repair, metabolism, and transcription [1]. In mammals, the sirtuin family comprises seven highly conserved proteins (SIRT1–7) that function as class III histone deacetylases and ADP-ribosyltransferases, linking cellular metabolic status to epigenetic and transcriptional regulation [1,2,3]. Dysregulation of SIRT activity has been implicated in a wide range of pathological conditions, including cancer, neurodegeneration, and metabolic disorders [1,4,5,6,7], underscoring the therapeutic potential of targeting sirtuins.

The human sirtuins share a conserved catalytic core of approximately 275 amino acids, consisting of a Rossmann-fold domain that binds NAD^+^/ADP-ribose and a zinc-binding domain stabilized by four conserved cysteine residues [8,9,10]. These domains are connected by flexible interdomain loops that form the active-site cleft [8,9,10,11,12,13,14,15,16]. The NAD^+^-binding region is organized into three functional pockets: the A-pocket accommodates the adenine-ribose moiety, the B-pocket accommodates the nicotinamide-ribose moiety, and the C-pocket accommodates the nicotinamide moiety of NAD^+^. Adjacent to these pockets, the extended C-site can provide an additional ligand-binding region, whereas the acetyl-lysine channel accommodates the substrate lysine side chain during catalysis [17,18]. Despite this conserved architecture, differences in non-conserved local residues, loop flexibility, pocket geometry, and electrostatic environment can influence ligand recognition across sirtuin isoforms. Ligand binding may also induce conformational rearrangements and transient secondary pockets that are not apparent in static protein structures. For example, ligand-induced expansion of the SIRT2 active site can generate a selectivity pocket exploited by isoform-selective inhibitors [19], while comparative studies of SIRT1–3 have shown that local residues and protein dynamics can contribute to differential inhibitor recognition [20]. Consequently, static docking can identify plausible interaction patterns but cannot by itself establish isoform selectivity [12,18].

We have previously reported the antitumor efficacy and toxicological profile of the benzimidazole-based small-molecule inhibitor BZD9L1 in colorectal cancer (CRC) models [21,22,23,24,25,26,27]. BZD9L1 was shown to inhibit SIRT1 and SIRT2 activity, suppressing cell viability, migration, survival, and angiogenesis through the induction of apoptosis in both in vitro and in vivo systems [22,24,25]. However, characterization of BZD9L1 has thus far been limited to SIRT1 and SIRT2 [21,28], and its interactions with the remaining sirtuin family members (SIRT3–7) remain largely unexplored. Comparative investigation of the potential interactions of BZD9L1 with other sirtuin isoforms may therefore provide testable hypotheses regarding its broader molecular recognition profile and inform subsequent experimental characterization.

Structure-based molecular modelling has been widely applied to characterize chemically diverse sirtuin modulators and investigate structural determinants of inhibitor potency and isoform selectivity [14,29,30,31]. Structure-based molecular modelling and docking approaches have previously been applied to characterize sirtuin–ligand interactions, particularly for SIRT1 and SIRT2 inhibitors [32,33,34]. Early in silico studies identified small-molecule SIRT2 inhibitors through virtual screening and docking within the NAD^+^-binding region, highlighting the feasibility of computational strategies for sirtuin targeting [35]. Subsequent molecular docking and molecular dynamics simulation studies demonstrated that ligand binding can induce local conformational rearrangements in the SIRT active site and that hydrogen-bonding networks and hydrophobic contacts within the NAD^+^-binding pocket are key determinants of binding affinity and selectivity [33,36]. More recent structure-based analyses have further emphasized the importance of active-site flexibility and conserved catalytic residues in governing inhibitor recognition across sirtuin isoforms [17,32]. Given the strong conservation of the sirtuin catalytic core [17,28,37], we hypothesized that BZD9L1 may interact with sirtuins beyond SIRT1 and SIRT2, particularly the less-characterized isoforms SIRT3–7. Among these, SIRT4 and SIRT7 remain poorly understood due to historically limited structural information [38]. Recent studies have revealed that SIRT4 possesses isoform-specific structural features, including a unique acyl-binding site and regulatory loop architecture that underlie its distinct substrate specificity, providing new opportunities for structure-based targeting [39,40].

In this study, we conducted a comparative structure-based evaluation of potential BZD9L1 binding modes and molecular interaction networks across all seven human sirtuin isoforms. X-ray crystal structures of SIRT1, SIRT2, SIRT3, SIRT5, and SIRT6 were integrated with homology models of SIRT4 and SIRT7. Molecular docking incorporating selected active-site side-chain flexibility was used to generate plausible binding conformations within the ADP-ribose-binding region. The resulting poses and predicted interaction patterns were compared across the seven isoforms to identify conserved and variable structural features. Together, this integrated analysis provides a comparative structural framework for investigating BZD9L1 recognition across the human sirtuin family and identifies testable interaction features for future mechanistic and structure-guided studies.

## 2. Results

### 2.1. Homology Modelling and Assessment of SIRT4 and SIRT7 Structures

**Figure 1:**
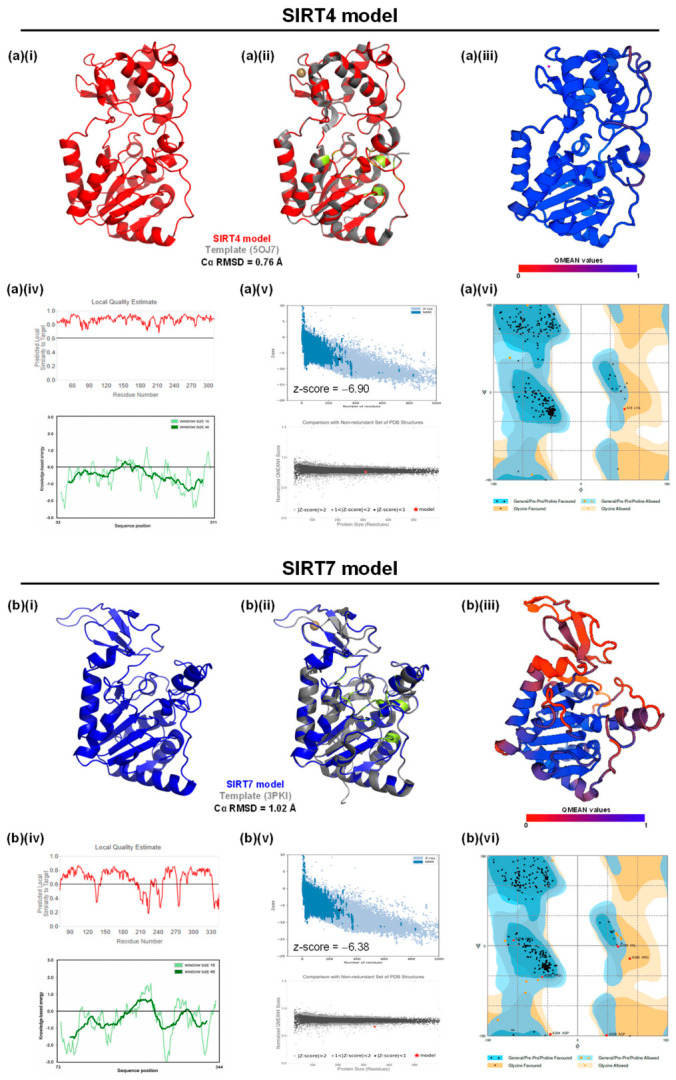
Homology models of (**a**) SIRT4 and (**b**) SIRT7. Homology models were (**i**) generated using SWISS-MODEL and (**ii**) superimposed onto their respective template structures. Models were evaluated by assessment of (**iii**) per residue quality, (**iv**) local QMEAN estimates and local model quality energy plot calculated against the average of 40-residue fragments (thick line) and 10-residue fragments (thin line), (**v**) the Z-score plot of all experimentally-determined protein chains in the PDB (light blue and dark blue dots represent structures determined via X-ray diffraction crystallography and NMR spectroscopy, respectively, and black dots represent model structures) and QMEAN4 score compared to QMEAN4 scores of similarly-sized experimental structures, and (**vi**) Ramachandran plot analysis. Proteins are shown in cartoon representation, with yellow-coloured regions in the template structures highlighting the backbone of cofactor-binding residues. Structures were superimposed using PyMOL.

Homology models of SIRT4 and SIRT7 were generated using the crystal structures 5OJ7 (chain A; Xenopus tropicalis NAD-dependent protein deacetylase) and 3PKI (chain A; human SIRT6), respectively, selected based on sequence identity (66.79% for SIRT4 and 42.75% for SIRT7) and conservation of residues within the cofactor-binding region (Figure 1a,b; Supplementary Figure S1a,b). The resulting Cα root-mean-square deviation (RMSD) values relative to the corresponding templates were 0.76 Å for SIRT4 and 1.02 Å for SIRT7 (Figure 1a(i,ii),b(i,ii); Table 1).

Despite the presence of several disordered regions in SIRT7, both models displayed favourable global Qualitative Model Energy ANalysis (QMEAN) scores and individual quality term Z-scores (Cβ, all-atom, solvation and torsion), particularly within the cofactor-binding region (Figure 1a(iii),b(iii); Table 1). Local quality assessment further indicated high per-residue reliability for residues comprising the ADP-ribose (ADPR)-binding site in both models (Figure 1a(iv),b(iv); Supplementary Figure S1c,d). The global QMEAN Z-scores of both models fell within the distribution of experimentally determined PDB structures of comparable size (Figure 1a(v),b(v); Table 1).

Ramachandran analysis showed that 98.6%, 1.1%, and 0.4% of SIRT4 residues were located within favoured, allowed, and outlier regions, respectively, whereas the corresponding values for SIRT7 were 94.1%, 3.7%, and 2.2% (Figure 1a(vi),b(vi); Table 1). The SIRT4 outlier K78 and the four SIRT7 outliers (A215, D234, R290, and D338) were mapped onto the corresponding model structures and were located outside the predicted ADPR-binding regions (Supplementary Figure S1e,f). The SIRT7 model additionally exhibited a global QMEAN Z-score of −2.67, a MolProbity score of 1.96, a clash score of 8.60, and two Cβ deviations (Table 1). Although these metrics indicate greater global structural uncertainty for SIRT7 than for SIRT4, the spatial separation of the Ramachandran outliers from the cofactor-binding region supports the use of the local pocket architecture for exploratory comparative docking.

Comparison with the corresponding AlphaFold Database predictions showed close structural agreement for the SIRT4 homology model, with an unpruned global Cα RMSD of 0.71 Å across 279 corresponding residues and a locally fitted ADPR-region Cα RMSD of 0.46 Å across 19 predefined residues (Supplementary Figure S1g–i). The comparison with SIRT7 showed greater global model-dependent differences, with an unpruned Cα RMSD of 5.35 Å across 272 corresponding residues, whereas the predefined ADPR-binding region showed closer local agreement, with a Cα RMSD of 1.00 Å across 19 residues (Supplementary Figure S1j–l). The mean AlphaFold pLDDT values for the predefined ADPR regions were 96.78 for SIRT4 and 92.62 for SIRT7. These comparisons indicated greater agreement within the predicted cofactor-binding regions than across the complete common modelled regions, particularly for SIRT7.

**Figure 2:**
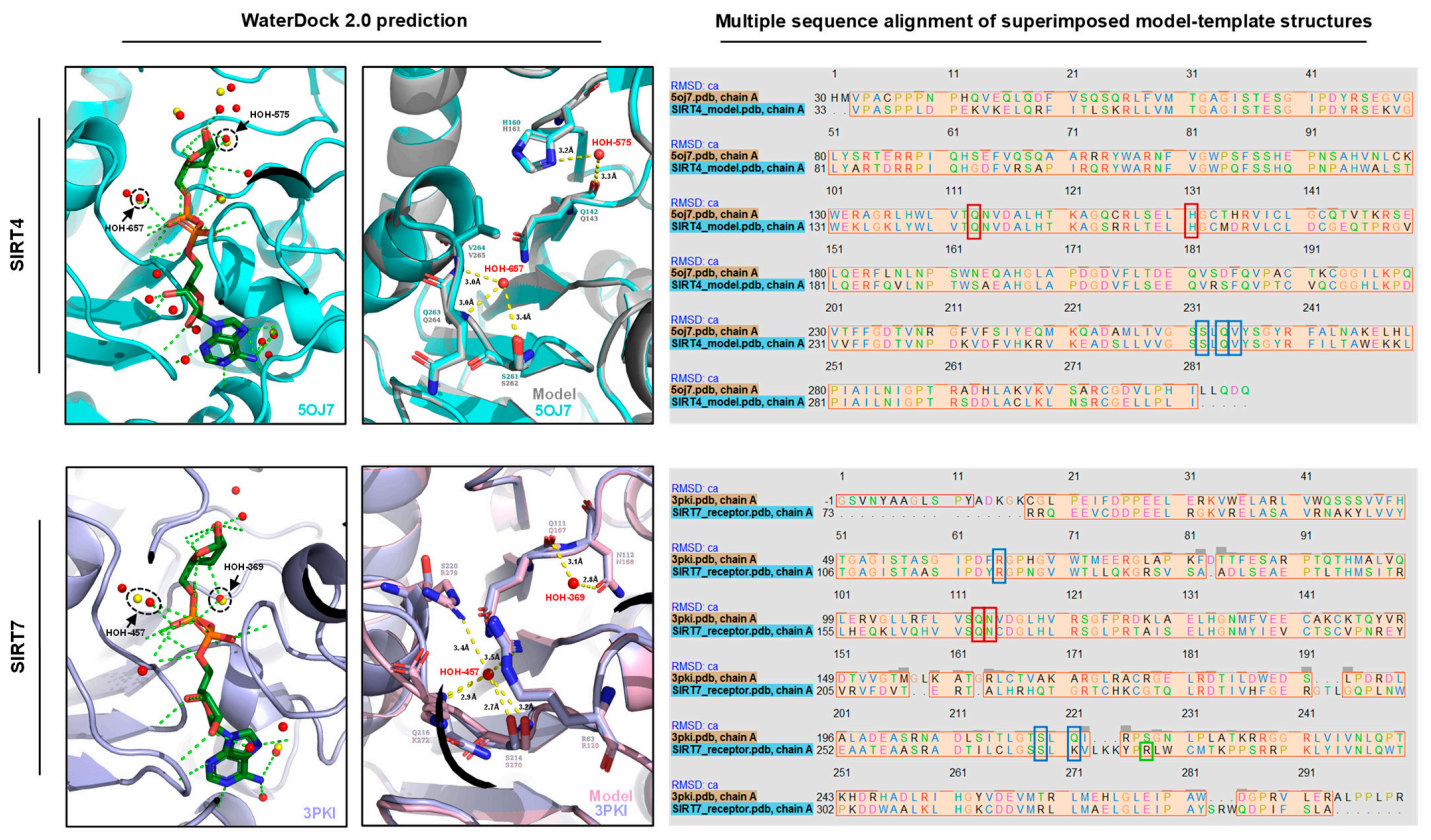
Location of potential water molecules in the active site of SIRT4 and SIRT7 models. WaterDock 2.0 was used to predict conserved water molecule positions in SIRT4 (PDB ID: 5OJ7) and SIRT7 (PDB ID: 3PKI) template structures. Conserved water molecules are shown for crystal structure water molecules (red spheres) that overlap with predicted water molecules (yellow spheres). Superimposition of model structures to their respective template structures shows excellent alignment of modelled and template residues interacting with potential crystal structure water molecules (red spheres). Sequence alignment between superimposed model-template structures indicates that the types of interacting residues in the templates are identical or have similar polarity to those in the models (shown in boxes). Residues interacting with crystal structure water molecules HOH-575 (SIRT4) and HOH-369 (SIRT7) are shown in red boxes. Residues interacting with crystal structure water molecules HOH-657 (SIRT4) and HOH-457 (SIRT7) are shown in blue boxes. An additional Arg residue in the SIRT7 model was positioned to form a predicted contact with HOH-457 and is shown in a green box. Proteins are shown in cartoon representation, with side chains and ADPR shown in licorice representation. Green dotted lines represent hydrogen bonds between ligand and water molecules. Yellow dotted lines represent hydrogen bonds between residues and water molecules. Structures were superimposed using PyMOL.

The SIRT4 and SIRT7 homology models therefore enabled extension of the comparative structure-based analysis to all seven human sirtuin isoforms. Nevertheless, homology modelling remains sensitive to local side-chain conformations, loop geometry, and water positioning, which may affect ligand poses and residue-level contact assignments (Figure 2; Supplementary Figure S7). The AlphaFold comparison does not validate the selected docking poses or predicted contacts. The SIRT4 and SIRT7 docking results were therefore retained as hypothesis-generating structural models requiring validation using experimentally determined structures or complementary biochemical and biophysical approaches.

### 2.2. Identification of Conserved Active-Site Water Molecules in SIRT4 and SIRT7 Models

The predictive performance of WaterDock 2.0 was first validated using SIRT1 and SIRT2 crystal structures by comparing predicted water molecule positions with those resolved experimentally (Supplementary Figure S2). In the SIRT4 model, WaterDock 2.0 accurately reproduced the positions of two conserved water molecules (HOH-575 and HOH-657) present in the template structure and interacting with equivalent residues in the modelled protein (Figure 2).

In the SIRT7 model, predicted water positions corresponding to HOH-369 and HOH-457 were geometrically compatible with hydrogen-bonding contacts involving semi-conserved residues and, in one case, an additional neighbouring residue (Figure 2). Predicted water molecule positions from both template structures were transferred into the corresponding homology models and retained for subsequent docking simulations and analyses.

In the SIRT7 model, the presence of R279 in place of S220 in the template also positioned the longer, positively charged arginine side chain to form an additional predicted hydrogen-bonding contact, while the semi-conserved Q216/K272 residue pair retained a comparable predicted polar contact (Figure 2).

### 2.3. Cofactor-Binding Sites Are Highly Conserved Across SIRT Isoforms

**Figure 3:**
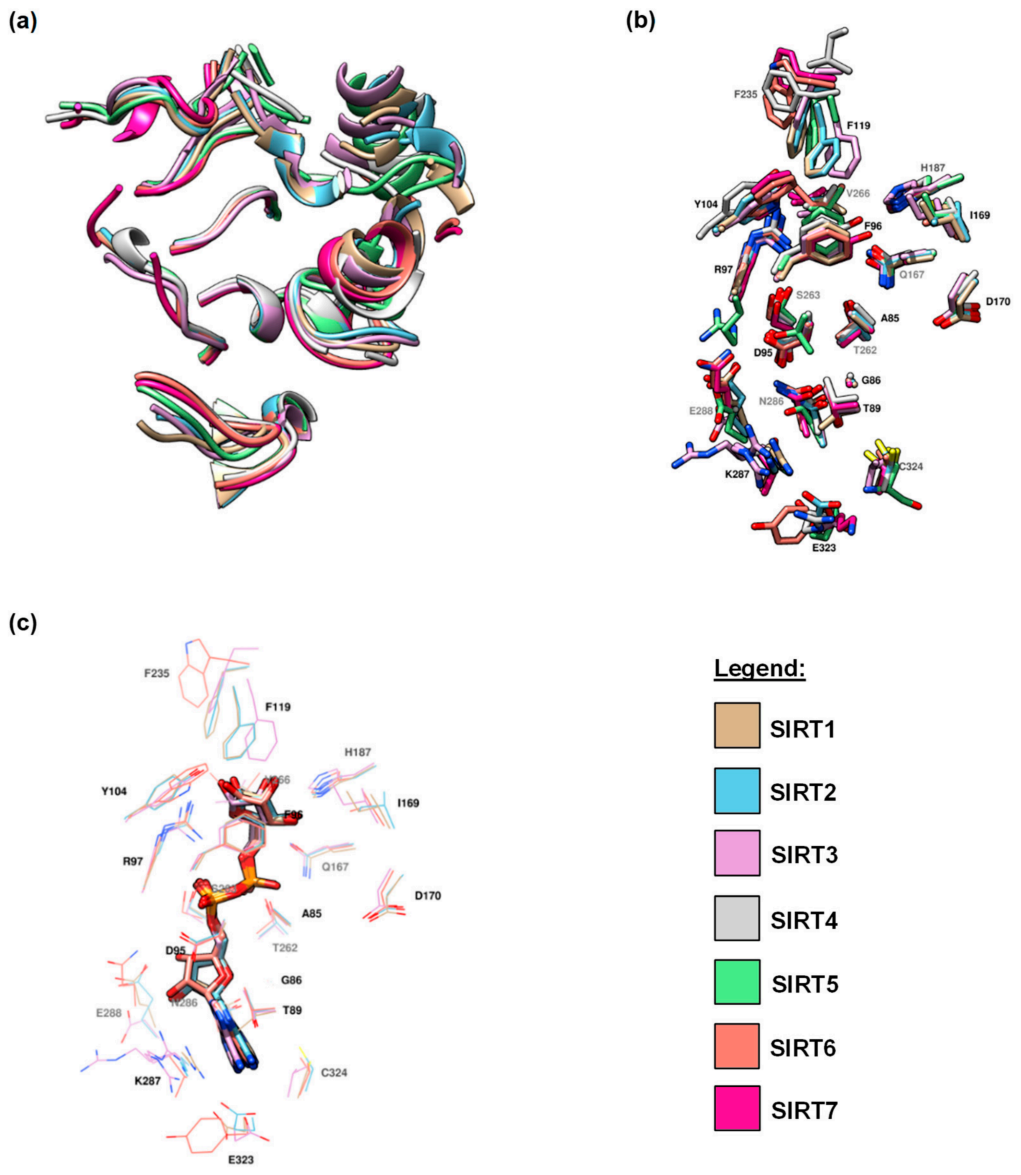
Conservation of the active sites of SIRT1-7 proteins. Structural superimposition of the crystal structures of SIRT1 (tan), SIRT2 (sky blue), SIRT3 (plum), SIRT5 (light green) and SIRT6 (salmon), and homology models of SIRT4 (light grey) and SIRT7 (deep pink) reveal a high level of conservation and structural alignment in the co-factor binding site for both (**a**) Cα backbone and (**b**) side chains. (**c**) The location and conformation of co-factor ADPR as well as the interacting amino acid residues are highly conserved in all SIRTS. Active site Cα backbone chains are shown in representation and side chains are shown in licorice. ADPR is shown in licorice and active site residue side chains are shown as lines in (**c**). Structural superimposition was performed using UCSF Chimera. Active site side chains were labelled using the amino acid numbering in the SIRT2 structure.

Structural superimposition of SIRT1–7 revealed a high degree of Cα backbone alignment and conserved side-chain positioning within the ADPR-binding region (Figure 3a,b). Inhibitor-bound structures showed no appreciable backbone deviation from the corresponding reference structures, whereas some activator-bound structures, particularly SIRT1 and SIRT5, displayed outward displacement of active-site loops (Supplementary Figure S3).

Consistent with this observation, the position of ADPR within the cofactor-binding site was highly conserved across all SIRT isoforms, exhibiting substantial spatial overlap (Figure 3c). Multiple sequence alignment of the structurally aligned SIRT receptors identified 19 ADPR-binding residue groups, comprising 12 identical, 3 conserved, 2 semi-conserved, and 2 non-conserved groups (Supplementary Figure S9). These residues were subsequently designated for side-chain flexibility in docking simulations involving SIRT4 and SIRT7.

### 2.4. Receptor Side-Chain Flexibility Improves Docking Accuracy of Benzimidazole Ligands

**Figure 4:**
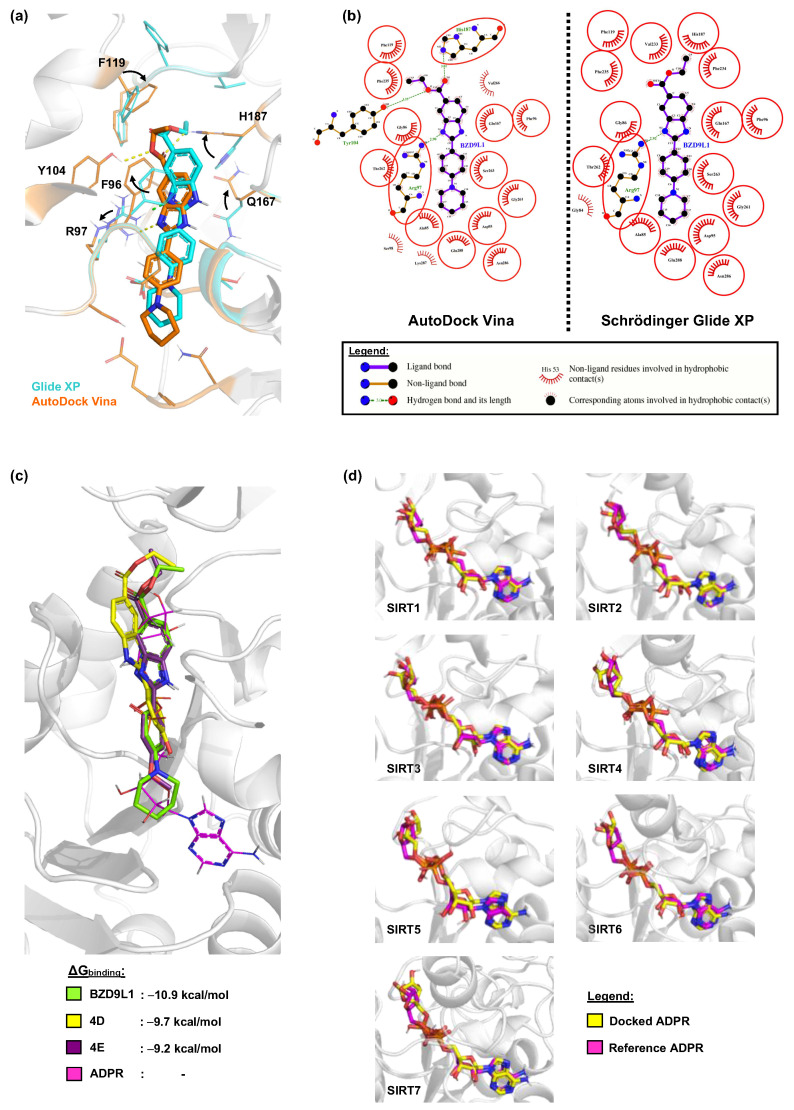
Amino acid residue side chain conformational flexibility in the molecular docking simulations. (**a**) Superimposition of BZD9L1 docked into the SIRT2 structure using AutoDock Vina (orange) and Schrödinger Glide XP (cyan). (**b**) Predicted interactions of BZD9L1 with SIRT2 using AutoDock Vina and Schrödinger Glide XP. The selected Schrödinger Glide XP pose contained one predicted hydrogen-bonding contact (green dotted lines) with SIRT2, whereas the selected AutoDock Vina pose (side chain rotation shown by black arrows) contained three predicted hydrogen-bonding contacts (yellow dotted lines) and exhibited a higher number of interactions. Red circles indicate residues predicted to contact BZD9L1 in both the selected AutoDock Vina and Schrödinger Glide XP poses. (**c**) Predicted docking poses and binding free energies to SIRT2 for BZD9L1 (green), 4D (yellow), and 4E (purple) upon binding with selected flexible side chains. Binding free energies were obtained from AutoDock Vina. (**d**) ADPR redocked into the prepared semi-flexible SIRT receptors using AutoDock Vina is shown in yellow, and the corresponding reference ADPR orientation is shown in magenta. For SIRT1, SIRT2, SIRT3, and SIRT6, the redocked poses were compared with ADPR resolved in the respective selected crystal structures. For SIRT5, ADPR was redocked into the prepared 6EQS receptor and compared with the ADPR orientation resolved in the structurally aligned SIRT5 structure 2B4Y, because 6EQS contains the ADPR-like inhibitor BV8 rather than ADPR. For the SIRT4 and SIRT7 homology models, the redocked poses were compared with ADPR from their respective template structures, 5OJ7 and 3PKI. Proteins are shown in cartoon representation, and residue side chains and ligands are shown in stick or line representation. Structural superimposition was performed using PyMOL, and protein–ligand contacts were visualized using LigPlot+.

AutoDock Vina and Schrödinger Glide predicted broadly similar orientations of BZD9L1 within the SIRT2 binding site (Figure 4a). In both selected docking poses, BZD9L1 was positioned to form a predicted hydrogen-bonding contact with R97. AutoDock Vina additionally predicted hydrogen-bonding contacts with Y104 and H187, associated with conformational adjustments of selected active-site side chains (Figure 4a). Overall, the selected AutoDock Vina pose contained predicted contacts with 18 residues, whereas the selected Schrödinger Glide pose contained predicted contacts with 17 residues (Figure 4b).

When the related benzimidazole analogues 4D and 4E, whose chemical structures are shown together with BZD9L1 in Supplementary Figure S4a, were docked into a fully rigid SIRT2 receptor, their predicted orientations were inverted relative to the selected BZD9L1 pose (Supplementary Figure S4b). These inverted poses did not reproduce the predicted hydrogen-bonding contacts identified in the selected BZD9L1–SIRT2 pose and were considered less structurally consistent with the previously reported structure–activity relationships of the three compounds. By contrast, allowing selected active-site side chains to adopt alternative conformations produced comparable orientations for BZD9L1, 4D, and 4E (Figure 4c).

Among the docking approaches evaluated, AutoDock Vina generated poses that were more consistent with the previously reported structure–activity relationships of BZD9L1 and the related compounds 4D and 4E, while also permitting conformational flexibility of selected active-site side chains (Figure 4c; Supplementary Figure S4c). AutoDock Vina was therefore selected to generate structurally comparable poses using the semi-flexible SIRT receptors. Redocking of ADPR into the corresponding SIRT receptors yielded RMSD values ranging from 1.067 to 1.368 Å relative to experimentally resolved or template-derived reference ADPR orientations, below the commonly applied 2.0 Å threshold for pose recovery (Figure 4d). These results supported the ability of the docking protocol to reproduce plausible cofactor-binding orientations but did not validate quantitative cross-isoform ranking of BZD9L1-predicted binding energies. The methodological factors described above may influence individual poses and residue-level contact assignments. The docking predictions were therefore interpreted qualitatively.

### 2.5. Predicted BZD9L1 Binding Poses Show Variable Interaction Patterns Across SIRT Active Sites

**Figure 5:**
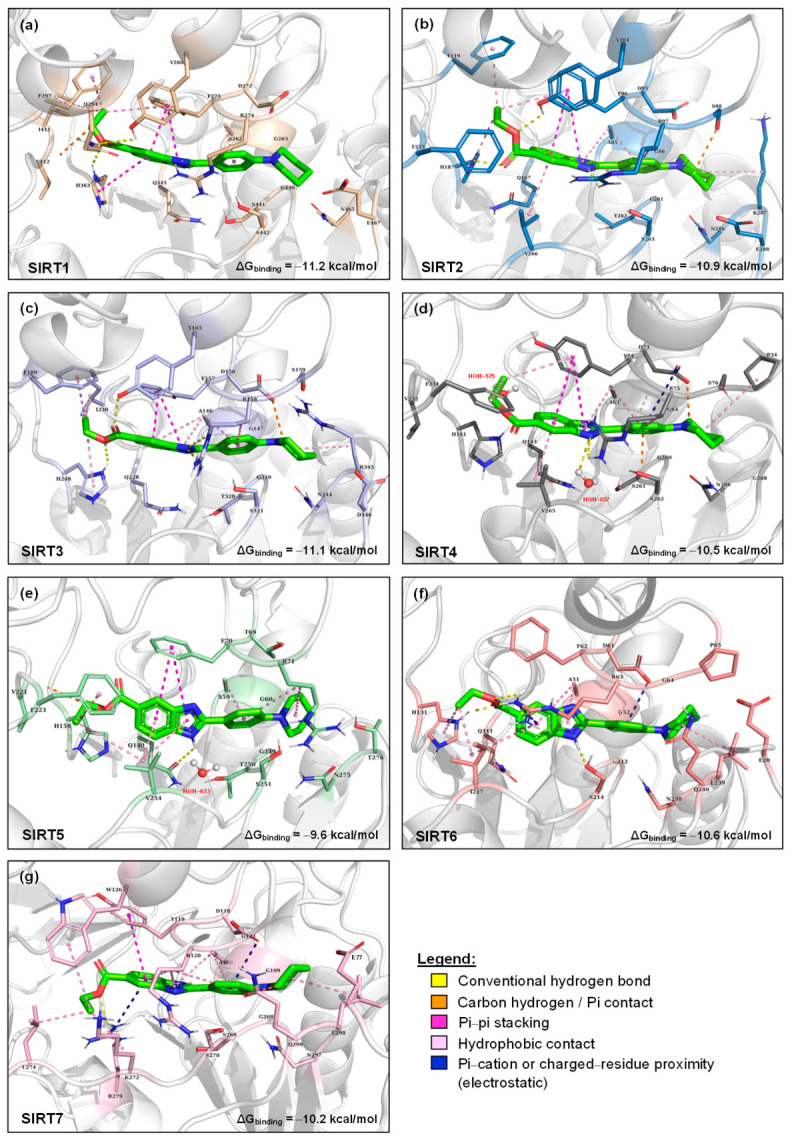
Predicted amino acid contacts in the selected BZD9L1 docking poses across SIRT1–7. Predicted interactions upon docking of BZD9L1 to (**a**) SIRT1, (**b**) SIRT2, (**c**) SIRT3, (**d**) SIRT4, (**e**) SIRT5, (**f**) SIRT6 and (**g**) SIRT7 identified using Discovery Studio and Ligplot+. Parameters were used for hydrogen bond length ≤ 3.50 Å and hydrophobic interactions within 4.0 Å from the ligand. Proteins are shown in cartoon representation. Residue side chains and BZD9L1 (in chartreuse) are shown in licorice representation coloured according to element: carbon in green, oxygen in red and nitrogen in blue. Active site water molecules are shown in ball-and-stick representation. Dotted lines indicate measured spatial proximities between BZD9L1 and nearby residues and do not, by themselves, denote assigned interactions. The colors of the dotted lines correspond to the predicted interaction types indicated in the figure legend: conventional hydrogen bond, carbon hydrogen/pi contact, pi–pi stacking, hydrophobic contact, and pi-cation or charged-residue proximity. Residues are labelled using the single-letter amino acid code followed by the residue number in graphics generated using PyMOL. Binding free energies (ΔGbinding) were predicted using AutoDock Vina v1.1.2 software.

The AutoDock Vina-predicted binding free energies for the selected BZD9L1 poses ranged from −9.6 to −11.2 kcal mol^−1^ across SIRT1–7 (Figure 5; Supplementary Table S1). The predicted interaction networks comprised hydrogen-bonding, π–π stacking, and other hydrophobic contacts, together with several pose-specific proximities to charged residues (Figure 5; Supplementary Table S2). The selected BZD9L1 poses exhibited modest variation in AutoDock Vina scores across the seven sirtuin isoforms (Supplementary Table S1). These scores are approximate outputs of the docking function and were not interpreted as experimentally determined binding free energies or quantitative rankings of cross-isoform affinity. Accordingly, the numerical differences were considered qualitatively, with greater emphasis placed on comparing the predicted ligand orientations and interaction patterns across the selected poses.

Predicted hydrophobic contacts involving the ethyl moiety of BZD9L1 and aromatic or aliphatic residues were identified across the selected SIRT docking poses. In the selected docking poses, the ester group of BZD9L1 was positioned to form predicted hydrogen-bonding contacts with histidine and tyrosine residues in SIRT1–3 and with arginine residues in SIRT6 and SIRT7. The benzimidazole and phenyl ring systems participated in predicted π-stacking contacts, while the imidazole nitrogen was positioned to contact arginine or other polar residues in an isoform-dependent manner. Inclusion of selected active-site water positions affected the predicted interaction networks in an isoform-dependent manner. Inclusion of selected active-site water positions affected the predicted interaction networks in an isoform-dependent manner. In the selected SIRT4 docking pose, a predicted contact involved the template-corresponding active-site water molecule HOH-657, which contributed to a predicted bridging interaction between BZD9L1 and surrounding residues and was associated with a pose located within the B-pocket (Figure 5; Supplementary Figure S7). By contrast, a potential water-mediated contact in the selected SIRT2 pose did not satisfy the predefined hydrogen-bonding geometry and was therefore not classified as a hydrogen bond in the tabulated interaction network. However, because the geometry of a selected docking pose represents an approximate static model, the possibility of a water-mediated hydrogen bond following local structural relaxation cannot be excluded. In the selected SIRT4, SIRT6, and SIRT7 docking poses, D73, D61, and D118, respectively, were located near BZD9L1. These geometries were recorded as predicted spatial proximities. Predicted π-ring-cation electrostatic contacts with R63 and R279 were identified in the selected SIRT6 and SIRT7 poses, respectively (Figure 5; Supplementary Table S2).

### 2.6. Conservation of Residues Predicted to Contact BZD9L1 Across the SIRT Family

**Figure 6:**
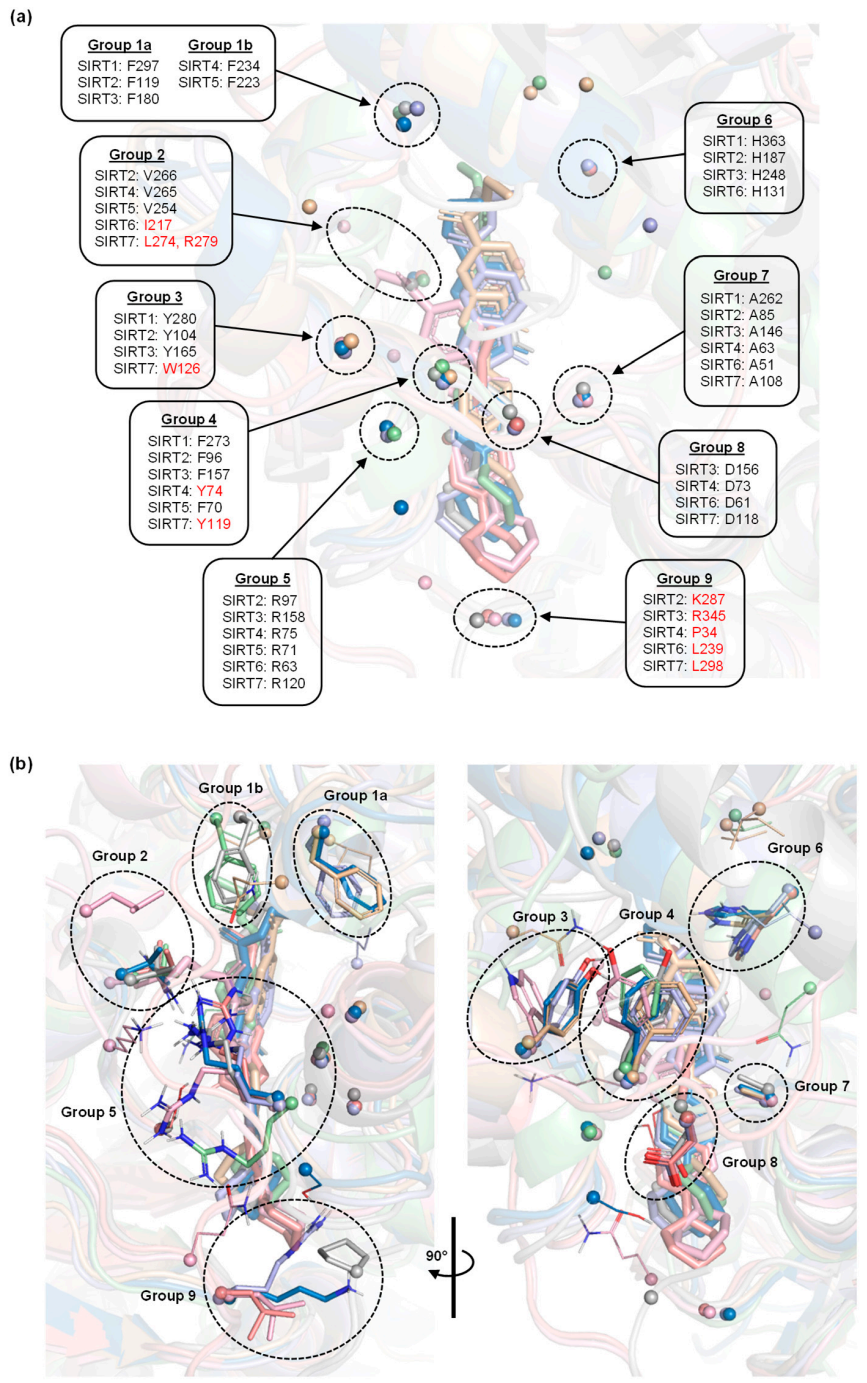
Classification of residues predicted to contact BZD9L1 in the selected SIRT1–7 docking poses. The active site backbone (Cα) of docked complexes was structurally superimposed using UCSF Chimera. (**a**) Conserved residues predicted to contact BZD9L1 were identified and grouped according to their structurally aligned positions. Non-identical residues from each group are highlighted in red. (**b**) Orientation and position of side chains from each group. Proteins are shown in cartoon representation, BZD9L1 and conserved side chains in licorice, and non-conserved side chains in wire representation. The Cα of relevant residues are shown as spheres. Different ligand-protein complexes correspond to SIRT1 (wheat), SIRT2 (sky blue), SIRT3 (light blue), SIRT4 (grey80), SIRT5 (pale green), SIRT6 (salmon), and SIRT7 (light pink). Residues are labelled with single-letter amino acid code followed by residue numbers.

Comparison of the selected docking poses revealed ≥75% conservation among residues predicted to contact BZD9L1 across human SIRT isoforms (Supplementary Figure S5). Structural superimposition identified nine residue groups exhibiting high Cα positional conservation within the binding site (Figure 6). While most groups comprised identical or conserved residues, Group 9 consisted of non-conserved residues.

**Figure 7:**
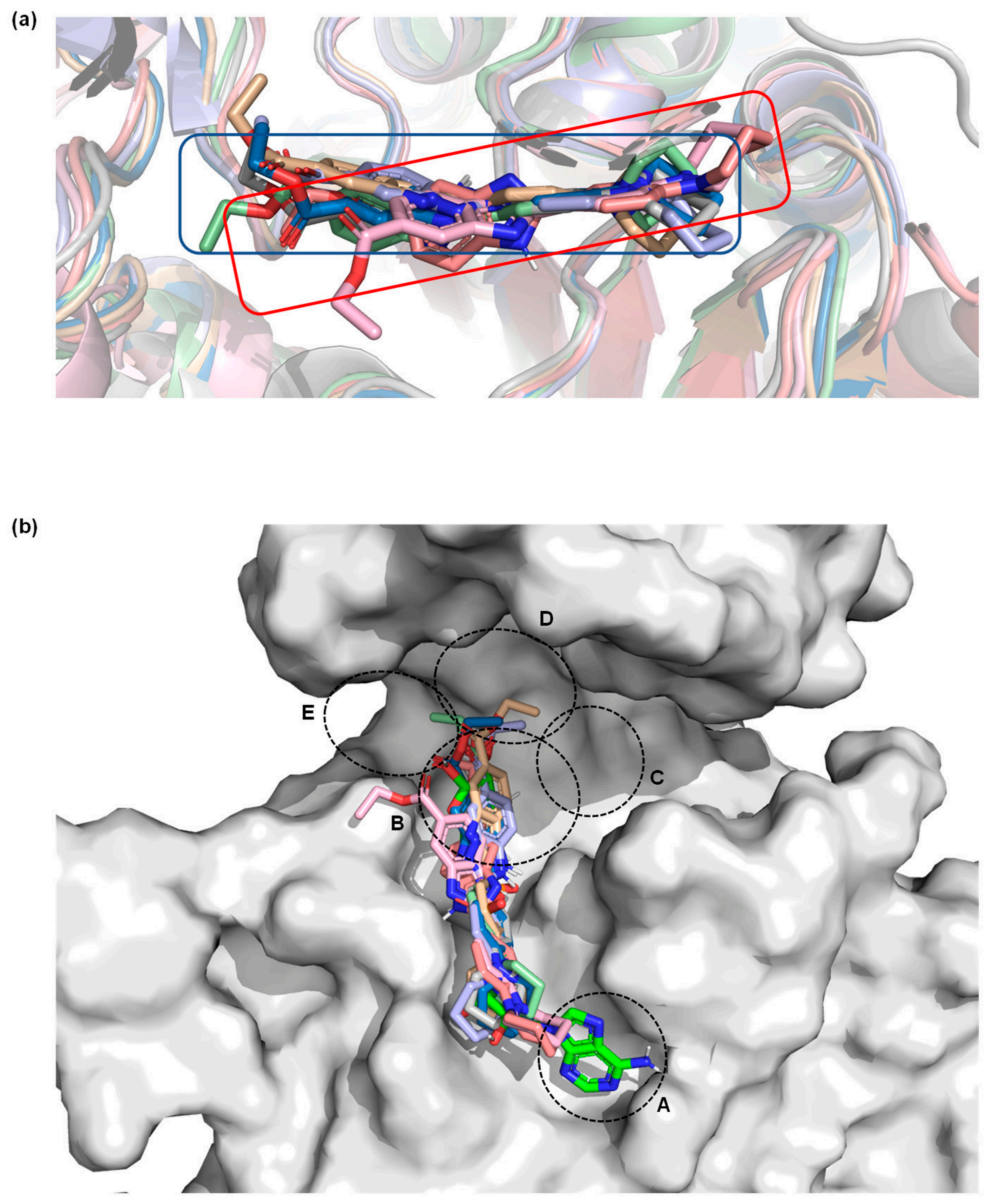
Predicted locations and orientations of BZD9L1 in the selected SIRT docking poses. (**a**) Superimposition of BZD9L1 docked into SIRT1 (wheat), SIRT2 (sky blue), SIRT3 (light blue), SIRT4 (grey80), SIRT5 (pale green), SIRT6 (salmon), and SIRT7 (light pink). Predicted binding modes of BZD9L1 in the active site of SIRTs were dominated by two docked poses (blue and red boxes). (**b**) Predicted orientations of BZD9L1 in the selected SIRT docking poses are shown relative to a grey surface representation of the SIRT2 crystal structure, with ADPR shown in green as a reference. Letters A, B, C, D, and E (black circles) indicate the A-pocket, which accommodates the adenine-ribose moiety of NAD^+^; the B-pocket, which accommodates the nicotinamide-ribose moiety; the C-pocket, which accommodates the nicotinamide moiety; the adjacent extended C-site, which can provide an additional ligand-binding region; and the acetyl-lysine channel, which accommodates the substrate lysine side chain, respectively. The protein is shown in cartoon representation and BZD9L1 in licorice. Superimposition was done using UCSF Chimera and visualisation was done using PyMOL.

Residue-level mapping of the selected BZD9L1 poses across the sirtuin family identified nine predicted interaction clusters associated with differences in ligand orientation, penetration depth, and contact patterns (Figure 6). Conserved hydrophobic and aromatic residues formed predicted π–π stacking and other hydrophobic contacts with the benzimidazole and ester-containing regions, whereas charged residues contributed to differences in predicted ligand orientation through electrostatic contacts (Figure 5; Supplementary Table S2). These cluster-level differences provide testable hypotheses for medicinal chemistry optimisation, including modification of ligand regions oriented toward electrostatic environments in the selected SIRT6 and SIRT7 poses and toward polar interaction networks in the selected SIRT1–3 poses. Despite these differences, the selected BZD9L1 docking poses showed broadly similar orientations across SIRT1–7. In the selected SIRT1–5 docking poses, BZD9L1 occupied the B-pocket and extended C-site of the ADPR-binding region, whereas the selected SIRT6 and SIRT7 poses spanned the B- and A-pockets (Figure 7).

### 2.7. BZD9L1 Treatment Alters Acetyl-SOD2 Levels in Colorectal Cancer Cells and Shows No Measurable SIRT5 Inhibition Under Cell-Free Assay Conditions

**Figure 8:**
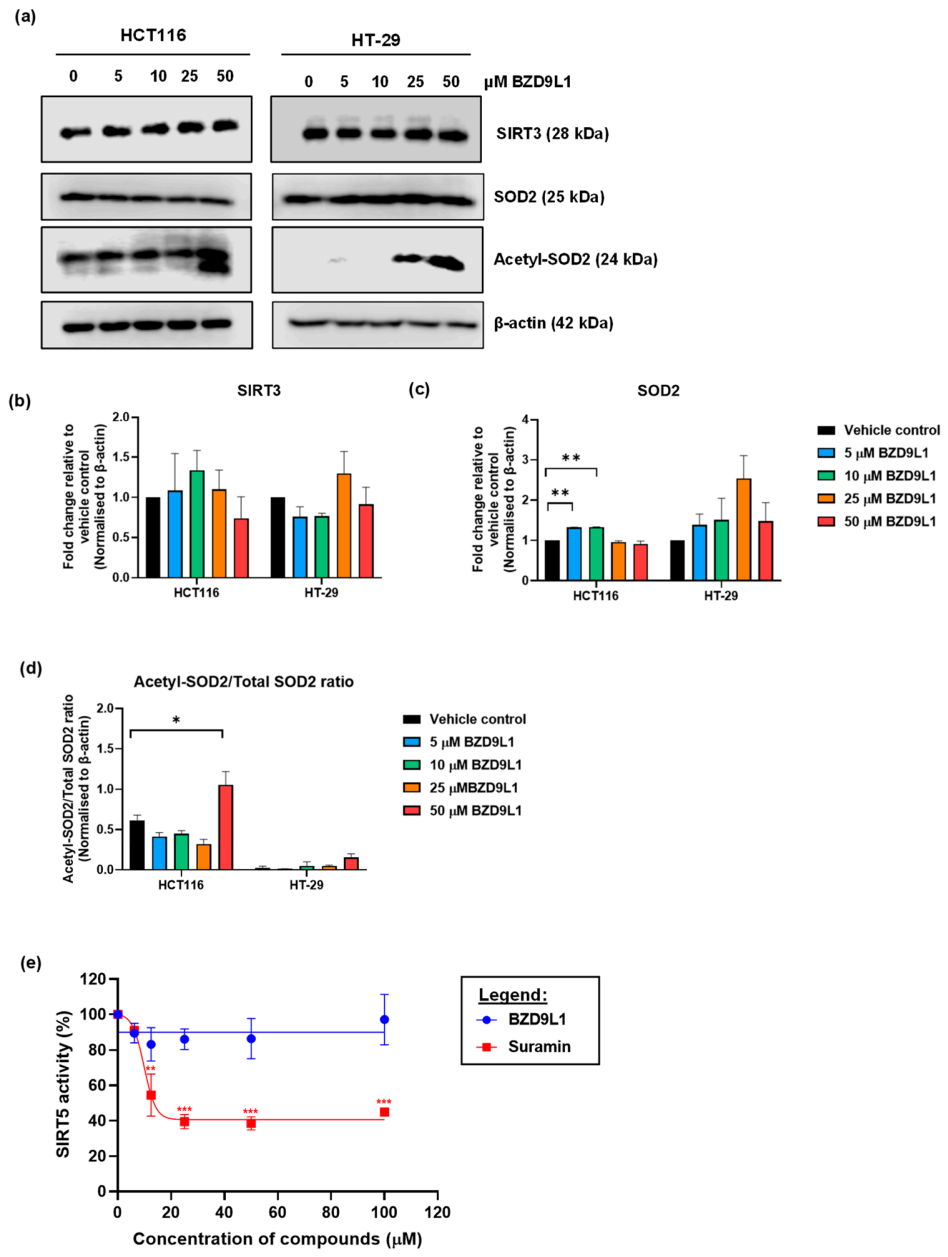
Cellular SIRT3-associated readout and cell-free SIRT5 enzymatic activity following BZD9L1 treatment. (**a**) Representative Western blots showing SIRT3, total SOD2, acetyl-SOD2, and β-actin levels in HCT116 and HT-29 cells treated with 0–50 µM BZD9L1. (**b**–**d**) Densitometric quantification of (**b**) SIRT3, (**c**) total SOD2, and (**d**) the acetyl-SOD2/total SOD2 ratio. Protein levels were normalized to β-actin and expressed relative to the corresponding vehicle control. (**e**) Cell-free SIRT5 enzymatic activity measured following treatment with BZD9L1 or suramin. Data are presented as mean ± SEM from three independent experiments. Densitometry data in panels (**b**–**d**) were analysed by one-way ANOVA followed by Bonferroni’s multiple-comparisons test, with the indicated BZD9L1 concentrations compared with the corresponding vehicle control. In panel (**e**), BZD9L1 and suramin were compared at each corresponding concentration using an unpaired, two-tailed Student’s *t*-test. Statistical significance is indicated by brackets or asterisks: * *p* < 0.05, ** *p* < 0.01, and *** *p* < 0.001.

BZD9L1 treatment did not markedly alter total SIRT3 protein expression in HCT116 or HT-29 cells under the conditions examined (Figure 8a,b). Changes in acetyl-SOD2 levels and the acetyl-SOD2/total SOD2 ratio were observed following BZD9L1 treatment, including an increase at 50 µM (Figure 8a,c,d). However, the response was not consistently concentration-dependent, particularly in HCT116 cells. These findings are consistent with altered SIRT3-associated deacetylation in a cellular context. Although this approach evaluates an endogenous intracellular SIRT3-associated substrate, it does not provide a direct enzymatic IC_50_ or demonstrate direct physical engagement of SIRT3. In a separate cell-free fluorometric assay, no measurable SIRT5 inhibition by BZD9L1 was detected under the conditions tested, whereas the kit-supplied suramin control reduced the assay signal (Figure 8e). Given the non-selectivity of suramin and its potential to affect fluorometric readouts, this reduction was interpreted only as evidence of assay responsiveness and not assay specificity. The absence of a separate enzyme-compound preincubation step may have limited assay sensitivity to slow-binding or time-dependent inhibition. Accordingly, the present findings do not exclude SIRT5 inhibition under alternative assay conditions. Because the SIRT3-associated cellular readout and SIRT5 enzymatic activity were assessed using different experimental systems, these results were not interpreted as a direct comparison of isoform selectivity.

## 3. Discussion

BZD9L1 is a benzimidazole-based small molecule previously characterized as a dual SIRT1/2 inhibitor, yet its interactions with the broader sirtuin family have remained insufficiently defined. Here, we combined structure-based modelling, molecular docking, and targeted experimental assessment to compare predicted BZD9L1 interaction patterns across all seven human sirtuin isoforms. The selected docking poses showed a broadly conserved orientation within the cofactor-binding region, together with differences in their predicted interaction networks.

Across the selected SIRT1–7 docking poses, BZD9L1 was predicted to contact 15–18 active-site residues that are highly conserved (~90%) among SIRT1–7 proteins (Figure 5; Supplementary Figure S5). The predicted BZD9L1 interaction networks comprised hydrogen-bonding, π-mediated, hydrophobic, and electrostatic contacts (Figure 5; Supplementary Table S2). These findings indicate that the benzimidazole scaffold of BZD9L1 exploits conserved architectural features of the sirtuin catalytic core while remaining sensitive to subtle differences in local pocket geometry and charge distribution. The predicted binding modes of BZD9L1 are consistent with earlier computational studies that localized inhibitor binding within the NAD^+^-binding cleft and emphasized the contribution of conserved hydrogen-bond networks and π-interactions to ligand stabilization [33,35,36]. Importantly, prior molecular dynamics simulation studies have shown that modest rearrangements of active-site side chains can substantially influence ligand orientation and predicted affinity, supporting the inclusion of receptor flexibility in docking simulations [31,32]. Our work extends these observations through a comparative structure-based analysis of predicted BZD9L1 binding modes across all seven human sirtuin isoforms.

The selected SIRT1–3 poses contained a greater number of predicted hydrogen-bonding contacts, whereas electrostatic and hydrophobic contacts contributed more prominently to the selected SIRT4, SIRT6, and SIRT7 poses (Figure 5a–g; Supplementary Table S2). Structural divergence was also associated with differences in predicted ligand positioning. In SIRT6 and SIRT7, the presence of a helical element in place of the α5 cofactor-binding loop may restrict local pocket adaptability and contribute to a shallower predicted binding pose spanning the B- and A-pockets (Figure 7; Supplementary Figure S6b). These structural differences provide a plausible explanation for the two principal binding orientations identified by docking and may inform future structure-guided investigation of isoform-dependent ligand recognition.

Interpretation of these predicted interaction patterns is nevertheless limited by the use of static receptor conformations with flexibility restricted to selected active-site side chains. Sirtuin inhibitor recognition can involve broader loop rearrangements, ligand-induced pocket formation, and isoform-dependent conformational dynamics that are not fully represented by this docking approach. In SIRT2, for example, ligand binding can induce formation of an extended selectivity pocket that is not evident in the unliganded active site [19]. Comparative analyses of SIRT1–3 have similarly demonstrated that non-conserved local residues and differences in protein dynamics can influence inhibitor recognition despite conservation of the catalytic core [20]. In relation to BZD9L1, these findings indicate that its predicted contacts within the conserved ADP-ribose-binding region may represent only part of the recognition mechanism, because broader conformational rearrangements and interactions with non-conserved adjacent residues could alter the predicted poses and their persistence in individual isoforms. The selected BZD9L1 poses therefore represent plausible structural hypotheses within the receptor conformations examined rather than definitive descriptions of isoform-selective binding. Molecular dynamics simulations, free-energy calculations, and experimental structural or binding studies will be required to determine whether the predicted orientations and contacts persist across the conformational ensembles of individual isoforms.

Experimental structural studies have identified distinct features of SIRT4, including an isoform-specific acyl-binding region, an additional access channel, and a regulatory loop extending toward the active site [39]. These experimentally observed features provide important context for interpreting the SIRT4 model and suggest that local conformational properties not fully represented by homology modelling may influence BZD9L1 recognition. Consideration of these features provides a structural context for interpreting the predicted SIRT4 pose (Figure 5d and Figure 7) and identifies specific regions for future experimental investigation.

Among all isoforms examined, SIRT5 displayed the weakest predicted interaction with BZD9L1, showing the least favourable binding free energy and the fewest stabilising contacts (Figure 5e; Supplementary Tables S1 and S2; Supplementary Figure S5). This prediction was concordant with the absence of measurable SIRT5 inhibition in vitro (Figure 8b). Reduced stabilisation in SIRT5 appears to arise from local structural constraints in the cofactor-binding region, including substitutions and/or absence of aromatic residues within conserved interaction clusters, which collectively limit optimal π-stacking and hydrogen-bonding networks (Figure 6 and Figure 7; Supplementary Figure S6b). In addition, the orientation of key charged residues may further restrict productive ligand accommodation (Supplementary Figure S6c). Together, these features provide a structural hypothesis for the less favourable predicted BZD9L1 interaction profile in SIRT5. The absence of measurable SIRT5 inhibition under the cell-free assay conditions was considered separately and does not validate the predicted binding pose or establish weak physical binding (Figure 5e and Figure 8b; Supplementary Tables S1 and S2).

Comparison with inhibitor-bound sirtuin structures provides additional context for the predicted binding modes of BZD9L1. Many reported sirtuin inhibitors engage the C-pocket and extended C-site, whereas the selected BZD9L1 docking poses primarily overlapped the ADPR-binding region, spanning the A- and B-pockets and, in some isoforms, extending toward the extended C-site (Figure 7; Supplementary Figure S8). The C-pocket and acetyl-lysine channel, together with portions of the extended C-site that were not occupied in individual selected poses, represent potential directions for chemical extension of the BZD9L1 scaffold. However, whether extension into these regions would improve affinity or isoform selectivity requires experimental evaluation and cannot be established from the present docking analysis. The predicted overlap of the selected BZD9L1 poses with the ADP-ribose-binding region is consistent with previous biochemical evidence that BZD9L1 acts as a reversible inhibitor of SIRT1 and SIRT2 and exhibits NAD^+^-competitive inhibition of SIRT2 [21]. However, the present docking analysis does not establish that BZD9L1 inhibits SIRT3–7 competitively or through the same kinetic mechanism. Whether BZD9L1 inhibits these additional isoforms and, if so, through comparable or distinct kinetic mechanisms remains to be determined experimentally.

Genetic variation within SIRT isoforms may influence inhibitor recognition and pharmacological responses by altering local pocket geometry, conformational dynamics, and ligand interaction networks. Although sirtuin inhibitors generally target the conserved NAD^+^-binding region, structural and mutational studies have demonstrated that non-conserved residues can contribute substantially to isoform-dependent inhibitor affinity and binding conformation. For example, residues involved in EX-527 binding have been shown to modulate inhibitor recognition across different SIRT isoforms [20,31]. The conserved and variable interaction clusters identified in the present study provide a structure-based framework for prioritizing naturally occurring or disease-associated variants that may alter BZD9L1 recognition. If validated experimentally, these relationships could inform the development of BZD9L1-related compounds with refined isoform-recognition profiles and support future investigation of molecular features associated with differential pharmacological response. By defining conserved and variable interaction features across SIRT1–7, this study establishes a structural foundation for the rational optimization of BZD9L1-related compounds and for future investigation of multi-isoform sirtuin modulation.

The computational analysis was complemented by experimental observations in colorectal cancer models. BZD9L1 treatment altered the acetylation of SOD2, an established endogenous substrate of SIRT3, in HCT116 and HT-29 cells (Figure 8a,d). The magnitude of the acetyl-SOD2 response varied across concentrations and cell lines, with an increase observed at 50 µM. Accordingly, these data were interpreted as consistent with altered SIRT3-associated deacetylation rather than as a conventional concentration-dependent response. This cellular measurement provides an indirect functional readout and not a direct assessment of SIRT3 enzymatic inhibition. Because no parallel cytotoxicity assay was performed under the same conditions, changes in cell viability or treatment-associated cellular stress cannot be excluded as contributors to the non-monotonic acetyl-SOD2 response, particularly in HCT116 cells.

Together with the previously reported activity of BZD9L1 against SIRT1 and SIRT2 [21], the present findings support continued investigation of BZD9L1 as a chemical scaffold for studying sirtuin recognition across distinct cellular compartments. However, SIRT4, SIRT6, and SIRT7 were evaluated computationally only and were not experimentally assessed in the present study. The predicted interactions with these isoforms should therefore be considered testable structural hypotheses rather than evidence of functional inhibition. Future studies should employ matched cellular and biochemical assay formats, orthogonal non-fluorescent approaches, and appropriate isoform-specific controls to determine whether BZD9L1 directly engages and modulates individual sirtuin isoforms. Such studies may also establish whether the predicted differences in local pocket architecture and non-conserved residues can guide the structure-based optimization of BZD9L1-related compounds.

In summary, this study provides a comparative structure-based assessment of plausible BZD9L1 binding modes across the human sirtuin family. The docking predictions identify conserved and variable interaction features that may influence ligand recognition, but they do not establish binding affinity or isoform selectivity. The experimental observations provide limited support for altered SIRT3-associated activity in cells and no measurable SIRT5 inhibition under the cell-free assay conditions used; however, the different assay formats preclude direct comparison between the two isoforms. These findings provide testable structural hypotheses regarding BZD9L1 recognition by individual sirtuin isoforms and may guide subsequent biochemical, biophysical, and structure-based evaluation of BZD9L1 and related benzimidazole derivatives.

## 4. Materials and Methods

### 4.1. Selection of SIRT Protein Structures

All X-ray diffraction (XRD) protein structures were obtained from the Research Collaboratory for Structural Bioinformatics (RCSB) Protein Data Bank (PDB; https://www.rcsb.org/) (assessed on 3 April 2019). Crystal structures of human SIRT1 (PDB ID: 4KXQ), SIRT2 (PDB ID: 3ZGV) [41], SIRT3 (PDB ID: 4BN4) [42], SIRT5 (PDB ID: 6EQS) [43], and SIRT6 (PDB ID: 3ZG6) [44] were selected for molecular docking analyses. Structure selection criteria included high-quality X-ray crystallographic resolution (≤2.5 Å), completeness of the active-site Cα backbone, and the presence of bound ADP-ribose (ADPR) or ADPR-like ligands in the absence of activator molecules.

Due to the lack of experimentally determined structures, SIRT4 and SIRT7 were modeled using homology-based approaches. All selected SIRT crystal structures were determined by X-ray crystallography and exhibited either high resolution (SIRT1, SIRT3, and SIRT5; ≤2.0 Å) or moderate resolution (SIRT2 and SIRT6; ≤2.5 Å), ensuring suitability for structure-based docking studies [45,46] Structures were additionally required to possess a fully resolved cofactor-binding region to enable accurate prediction of ligand binding poses. For SIRT5, the only available ADPR-bound structure (PDB ID: 2B4Y) displayed incomplete resolution within the cofactor-binding site [14]. Consequently, an alternative structure containing a bound ADPR-like inhibitor (BV8; PDB ID: 6EQS) with a fully resolved active site was selected. Ligand identity can influence SIRT active-site architecture, with activator binding being associated with isoform-dependent conformational changes, including widening of the active-site region in some structures, whereas the examined inhibitor-bound structures generally retained a closed conformation (Supplementary Figure S3). Therefore, only structures lacking bound activators were selected for the docking analysis. No explicit solution-pH-dependent standardization of receptor protonation states was performed. Hydrogen atoms were added during structure preparation, and the receptor structures retained the protonation states assigned during the recorded preparation procedure. The catalytic histidine was represented as HIP in SIRT1, SIRT3, SIRT4, SIRT6, and SIRT7, HID in SIRT5, and HIE in SIRT2. Momany–Rone partial charges were assigned to the receptor structures before conversion to the docking input format. Consequently, differences in receptor protonation and histidine tautomeric states represent a limitation of the cross-isoform comparison.

As BZD9L1 has been reported to occupy the ADPR-binding site within the SIRT2 active site [21], protein structures containing ADPR or ADPR-mimetic ligands were selected to ensure consistency with experimentally supported binding modes and to maintain a closed active-site conformation suitable for docking analyses. The selected SIRT5 structure (PDB ID: 6EQS) was therefore selected for molecular docking due to its fully resolved cofactor-binding region and presence of an ADPR-like ligand.

### 4.2. Homology Modelling

Amino acid sequences of SIRT4 (UniProt ID: Q9Y6E7) and SIRT7 (UniProt ID: Q9NRC8) were obtained from The Universal Protein Resource Knowledgebase (UniProtKB) (https://www.uniprot.org/) (assessed on 3 April 2019). Three-dimensional (3D) template crystal structures for modelling were then identified through the Standard Protein Basic Local Alignment Search Tool (BLASTp) (https://blast.ncbi.nlm.nih.gov/) (assessed on 3 April 2019) utilising the PDB protein database and the Blastp (protein BLAST) algorithm [47]. Template and query sequences were then subjected to multiple sequence alignment using Clustal Omega version 1.2.4 webserver (https://www.ebi.ac.uk/Tools/msa/clustalo/) (assessed on 3 April 2019) [48,49] for identification of conserved sequences, substitutions, and gaps to assess template quality. Homology models of SIRT4 and SIRT7 were produced using the SWISS-MODEL server (https://swissmodel.expasy.org/) (assessed on 3 April 2019) [50,51,52,53,54,55]. The quality and reliability of generated models were assessed using the (1) SWISS-MODEL Structure Assessment Tools (https://swissmodel.expasy.org/assess) (assessed on 11 August 2019) and the Protein Structure Analysis web server (ProSA-web) (https://prosa.services.came.sbg.ac.at/prosa.php) (assessed on 11 August 2019) [56,57], (2) structural backbone root-mean-square deviation (Cα RMSD) using PyMOL 2.3 (Schrödinger, New York, NY, USA), and (3) Ramachandran plot analysis using RAMPAGE (http://mordred.bioc.cam.ac.uk/~rapper/rampage.php) (assessed on 11 August 2019) [58]. Ramachandran-outlier residues identified by the SWISS-MODEL Structure Assessment Tools (https://swissmodel.expasy.org/) (assessed on 11 August 2019) and MolProbity v4.4 were mapped onto the SIRT4 and SIRT7 homology models using PyMOL. Their spatial locations were compared with the predicted ADPR-binding residues identified through structural alignment with the corresponding template structures. The SIRT4 and SIRT7 homology models were additionally compared with the corresponding human AlphaFold Database predictions, AF-Q9Y6E7-F1-v6 and AF-Q9NRC8-F1-v6, respectively. The models were structurally superimposed in PyMOL using corresponding Cα atoms over their common modelled regions, comprising residues 33–311 for SIRT4 and residues 73–344 for SIRT7. Global Cα RMSD values were calculated without iterative outlier rejection (cycles = 0). Separate local Cα fits were performed using the 19 predefined residues lining or structurally corresponding to the ADPR-binding region of each isoform. Residue-level pLDDT values were extracted from the B-factor field of the AlphaFold coordinate files. Because pLDDT is specific to AlphaFold predictions, it was not used as a direct quality measure for the SWISS-MODEL homology models.

### 4.3. Identification of Binding Sites and Amino Acid Residues with Flexible Side Chains

The co-factor binding sites in the SIRT structures were determined through the literature and protein databases [59,60]. The binding site backbone chain (Cα) of selected SIRT crystal structures or homology models was then structurally superimposed using MatchMaker and sequence-aligned via the Match ⟶ Align feature in UCSF Chimera version 1.14 (University of California, Berkeley, CA, USA) [61] to identify and select active site residues. All residues involved in binding were used to define the molecular docking grid box location and size. Amino acids within these grid boxes were inspected using Discovery Studio 4.0 (Accelrys Software Inc., San Diego, CA, USA) to identify relevant residues with rotatable side chains (e.g., Arg, Phe, His, etc.) for flexible side chain docking. The complete isoform-specific flexible residue sets and the grid-box centres and dimensions used for ADPR redocking and BZD9L1 docking are provided in Supplementary Table S3. Flexible-residue sets were defined separately for the two ligands, and all residues not included in the specified flexible sets were retained as part of the rigid receptor.

### 4.4. Identification and Prediction of Conserved Water Positions at the SIRT Cofactor-Binding Site

Available X-ray diffraction (XRD) structures for each SIRT isoform were structurally superimposed and visualised using UCSF Chimera v1.14 (Chimera; University of California, CA, USA) (http://www.rbvi.ucsf.edu/chimera/, 28 July 2026) to identify recurrent water positions within the cofactor-binding region. Experimentally resolved water molecules were considered conserved and retained for docking when they occupied corresponding positions among the aligned structures and formed hydrogen-bonding contacts, defined by a donor-acceptor distance of ≤3.4 Å, with at least two distinct residues surrounding the cofactor-binding site. The identities and physicochemical properties of the corresponding water-coordinating residues were also examined to assess conservation of the local interaction environment. Water molecules that did not satisfy these positional and interaction criteria were removed during receptor preparation.

Conserved active-site waters can contribute to ligand recognition by mediating protein–ligand contacts and were therefore considered during receptor preparation and docking [62,63,64,65,66,67]. Typically, the positions of important waters are acquired from high-resolution crystal structures, such that comparisons across related protein crystal structures can be used to identify conserved water molecules [68,69,70]. Because experimentally resolved water molecules were unavailable in the SIRT4 and SIRT7 homology models, potential water positions within their cofactor-binding regions were predicted using the bridging-water algorithm implemented in WaterDock 2.0 (https://github.com/bigginlab/WaterDock-2.0) (assessed on 20 November 2022) [71]. Docking grids encompassing the cofactor-binding regions were defined using AutoDockTools v1.5.6 (The Scripps Research Institute, La Jolla, CA, USA) [72], and WaterDock 2.0 was applied using AutoDock Vina v1.1.2 (The Scripps Research Institute, USA) [73]. The resulting water-containing models were structurally aligned with their respective template crystal structures using UCSF Chimera and examined using Discovery Studio.

Predicted water positions in the SIRT4 and SIRT7 models were retained only when they overlapped with, or occupied spatially corresponding positions to, experimentally resolved waters in the respective template structures and were geometrically compatible with hydrogen-bonding contacts involving identical, conserved, or physicochemically similar surrounding residues. These criteria were applied to select template-consistent water positions for inclusion in the subsequent receptor-preparation and docking procedures.

The predictive performance of WaterDock 2.0 was evaluated by applying the same procedure to desolvated crystal structures and comparing the predicted water positions with experimentally resolved waters in the corresponding XRD structures. Potential hydrogen-bonding contacts were evaluated using a maximum donor–acceptor distance of 3.50 Å, consistent with previous protein–ligand interaction studies [72,73]. Contacts with donor-acceptor distances of ≤2.50 Å, 2.50–3.20 Å, and 3.20–3.50 Å were classified as strong, moderate, and weak hydrogen-bonding geometries, respectively [74,75].

### 4.5. Protein/Receptor Structure Preparation

All protein structures were prepared by removing co-crystallized ligands and water molecules that did not satisfy the predefined retention criteria. Hydrogen atoms were then added to the protein structures. Receptor protonation states were not standardized across isoforms. During energy minimization, positional restraints were applied to the protein heavy atoms and retained water oxygen atoms, whereas water hydrogen atoms and the hydroxyl (-OH) groups of binding-site serine, threonine, and tyrosine residues, as well as the thiol (-SH) groups of binding-site cysteine residues, remained unrestrained. The retained water molecules were included as part of the receptor in all corresponding docking simulations. Their oxygen atoms were maintained at the selected conserved or template-corresponding positions, while their hydrogen atom orientations were optimized during energy minimization. All structures were parameterized using the Chemistry at HARvard Macromolecular Mechanics (CHARMM) force field and energy-minimized using the steepest-descent algorithm for up to 2000 steps or until an energy-gradient convergence criterion of ≤0.1 kcal mol^−1^ Å^−1^ was reached [76,77]. Momany–Rone partial charges were assigned to all protein structures before molecular docking [78].

### 4.6. Ligand Preparation

The chemical structures of BZD9L1 and its analogues 4D and 4E were constructed using ChemSketch v2018.2.1 (Advanced Chemistry Development Inc., Toronto, ON, Canada). Compounds 4D and 4E, previously reported by Yoon et al. [21], contain hydroxyl (–OH) and methoxy (–OCH_3_) substitutions, respectively, at the piperidinyl moiety of BZD9L1 and exhibited reduced in vitro inhibitory activity against SIRT2 relative to the parent compound. These compounds were therefore included as comparative ligands to validate the docking protocol through assessment of predicted binding free energies. The structure of ADP-ribose (ADPR) was retrieved from the RCSB PDB Ligand Expo (PDB Chemical Component ID: AR6; https://www.rcsb.org/ligand/AR6) (assessed on 25 November 2019). All ligands were converted to 3D geometries and subjected to energy minimization using Discovery Studio with the CHARMM force field. Alternative pH-dependent protonation and tautomeric states were not enumerated. Ligand PDBQT files were generated using AutoDockTools with its default charge-assignment settings. Gasteiger partial charges were assigned during PDBQT ligand preparation.

### 4.7. Molecular Docking Simulations

Ligand and receptor input files for molecular docking were prepared using AutoDockTools and Maestro version 11.9 (Schrödinger, USA). Docking simulations were performed using AutoDock Vina and Schrödinger Glide XP (Schrödinger, USA) with conformationally flexible ligands and semi-rigid receptors. Where applicable, selected active-site side chains were treated as flexible to permit local conformational adjustment during docking. Retained water molecules were included as part of the corresponding receptor structures. The grid-box parameters and flexible-residue sets were defined separately for BZD9L1 docking and ADPR redocking and are provided in Supplementary Table S3. Residues not included in the specified flexible sets were retained as part of the rigid receptor.

Candidate BZD9L1 poses were evaluated collectively according to the following criteria: (i) placement within the ADP-ribose-binding region; (ii) spatial consistency with the cofactor-binding orientation supported by ADPR redocking; (iii) predicted contacts with conserved or structurally corresponding active-site residues; (iv) AutoDock Vina–predicted binding energies; and (v) absence of substantial steric clashes or geometrically implausible contacts. Water-mediated contacts were considered supportive only when the retained water position satisfied the predefined positional and hydrogen-bonding criteria; their presence alone was not used to select a representative pose. Representative poses were therefore selected by considering the structural and scoring criteria collectively rather than solely based on the most favourable predicted binding energy. Molecular interactions within the selected poses were analysed using PyMOL, Discovery Studio, and LigPlot+ version 1.4 (EMBL-EBI, UK) [50].

Convergence of the docking simulations was assessed by progressively increasing the sampling level and examining the reproducibility of the top-ranked ligand orientation and predicted binding energy. For docking BZD9L1 to the SIRT receptors, AutoDock Vina achieved convergence only at exhaustiveness settings of ≥100. Accordingly, an exhaustiveness setting of 100 was used for the reported calculations. A single AutoDock Vina invocation was performed for each reported ligand–receptor condition. No user-defined random seed was specified; AutoDock Vina therefore generated the seed automatically for each calculation. By contrast, all Schrödinger Glide XP calculations achieved convergence using the default sampling parameters, and no further adjustment was required.

The ability of the docking protocol to recover plausible cofactor-binding orientations was assessed by redocking ADPR into each SIRT receptor. For SIRT1, SIRT2, SIRT3, and SIRT6, RMSD values were calculated relative to ADPR resolved in the corresponding selected crystal structures. For SIRT5, ADPR was redocked into the prepared 6EQS receptor and compared with ADPR resolved in the structurally aligned SIRT5 structure 2B4Y. For the SIRT4 and SIRT7 homology models, RMSD values were calculated relative to ADPR from the respective template structures, 5OJ7 and 3PKI. The corresponding ADPR grid-box parameters and flexible-residue sets are reported separately from those used for BZD9L1 in Supplementary Table S3. Root-mean-square deviation (RMSD) values between the crystallographic or template-derived ADPR orientation and the corresponding redocked pose were calculated using DockRMSD (https://zhanggroup.org/DockRMSD/) (assessed on 21 November 2022) [79]. AutoDock Vina-predicted binding energies, reported in kcal mol^−1^, were used for pose selection and qualitative comparison. Because these values are empirical outputs of the docking scoring function and may be affected by receptor-dependent systematic differences, they were not interpreted as experimentally determined binding free energies or quantitative rankings of cross-isoform affinity.

### 4.8. Cell Culture

Colorectal carcinoma HCT 116 (CCL-247) and colorectal adenocarcinoma HT-29 (HTB-38) cell lines were purchased from American Type Culture Collection (ATCC) (Rockwell, Milwaukee, WI, USA). Cells were cultured in Roswell Park Memorial Institute (RPMI) 1640 medium (Nacalai Tesque, Kyoto, Japan) supplemented with 10% foetal bovine serum (Tico Europe, Amstelveen, The Netherlands), 100 units/mL penicillin (Biowest, Bradenton, FL, USA) and 100 units/mL streptomycin (Biowest, USA) under standard conditions (37 °C, 5% CO_2_).

### 4.9. Cell Treatment

BZD9L1 was synthesised as previously described [11]. BZD9L1 was prepared as a 20 mM stock solution in dimethyl sulfoxide (DMSO) (Nacalai Tesque, Japan), aliquoted, and stored at −20 °C protected from light. For the 5–50 µM dose–response experiment, DMSO was maintained at 0.25% (*v*/*v*) in all treatment and vehicle-control groups. Cells were plated and allowed to adhere for 24 h prior to treatment with BZD9L1 or vehicle control. Aqueous DMSO was used as the vehicle control. Media and treatments were renewed every three days. A parallel cell-free fluorometric SIRT3 assay was not performed because the intrinsic fluorescence of BZD9L1 overlapped with the excitation and emission range of the available assay platform, potentially confounding signal interpretation. An endogenous cellular substrate was therefore examined as an alternative functional readout.

### 4.10. Western Blot

Cellular protein was extracted using 8 M urea lysis buffer supplemented with protease inhibitors (Nacalai Tesque, Japan) and was quantified using a Protein Assay BCA Kit (Nacalai Tesque, Japan). Protein extracts were resolved via 10% bis-acrylamide gel electrophoresis at a constant voltage of 150 V for 75 min. Protein samples were then transferred to an AmershamTM HybondTM P 0.45 µm PVDF Membrane (GE Healthcare Life Science, Buckinghamshire, UK) and blocked with 5% skimmed milk in TBST for 1 h. The membrane was probed with primary antibodies: β-actin (Sigma-Aldrich, St. Louis, MO, USA, Cat#096 M4855 V, mouse monoclonal, 1:5000 dilution), SOD2 (GeneTex, Irvine, CA, USA, Cat#GTX116093, rabbit polyclonal, 1:1000 dilution), SOD2/MnSOD (acetyl K68) [EPVANR2] (Abcam, Cambridge, UK, Cat#ab137037, rabbit monoclonal, 1:500 dilution), and SIRT3 (D22A3) (Cell Signaling Technology, Danvers, MA, USA, Cat#5490, rabbit monoclonal, 1:1000 dilution) at 4 °C overnight. Subsequently, the membrane was probed with anti-mouse IgG, HRP-linked (Cell Signaling Technology, USA, Cat#7076, horse polyclonal, 1:5000 dilution) or anti-rabbit IgG, HRP-linked (Cell Signaling Technology, USA, Cat#7074, goat polyclonal, 1:5000 dilution) secondary antibodies at room temperature for 1 h. The membrane was incubated with Chemi-Lumi One Super substrate (Nacalai Tesque, Japan) for 1 min and was scanned with C-DiGit^®^ Chemiluminescence Blot Scanner (LI-COR Biosciences, Lincoln, NE, USA). Densitometry analysis was performed using the Image Studio Lite version 5.2 software (LI-COR Biosciences, USA).

### 4.11. SIRT5 Activity Assay

The effect of BZD9L1 on SIRT5 enzymatic activity was evaluated using the SIRT5 Deacetylase Fluorometric Assay Kit (Abcam, UK) based on the manufacturer’s protocol. Briefly, appropriate combinations of reaction mixture (distilled water, SIRT5 Assay Buffer, Fluoro-Substrate Peptide or Fluoro-Deacetylated Peptide, and NAD) were added into the wells of a microtiter plate. The wells were then supplemented with BZD9L1, the kit-supplied suramin as a positive control, or DMSO as the vehicle control, followed by the developer solution. The reaction was initiated upon addition of recombinant SIRT5, and the microplate incubated at room temperature for 1 h on an orbital shaker. No separate enzyme-compound preincubation step was performed. Fluorescent intensity was determined by reading the samples with 480 nm excitation and 520 nm emission in a Varian Cary Eclipse Fluorescence Spectrophotometer (Agilent Technologies, Santa Clara, CA, USA).

### 4.12. Statistical Analysis

GraphPad Prism 8.0 software (GraphPad, Boston, MA, USA) was used to analyse all experimental data. The student’s *t*-test was used to compare mean values between two datasets. Analysis of variance (ANOVA) was used to compare mean values among three or more datasets. Bonferroni’s post-test was used to compare any two datasets among three or more sets. Statistical significance was indicated by * where *p* < 0.05, ** where *p* < 0.01, *** where *p* < 0.001 and **** where *p* < 0.0001. All error bars depict the standard error of the means (SEM).

## 5. Conclusions

This study compared plausible binding modes of BZD9L1 within the ADP-ribose-binding regions of human SIRT1–7. The selected docking poses showed broadly conserved orientations but differences in their predicted hydrogen-bonding, π-mediated, and hydrophobic contact patterns. Since docking scores provide qualitative predictions rather than experimental measurements of binding affinity, the present analysis does not establish differential affinity or isoform selectivity.

BZD9L1-induced changes in SOD2 acetylation were consistent with altered SIRT3-associated activity in a cellular context, whereas no measurable SIRT5 inhibition was observed under the cell-free assay conditions tested. Due to these assays differing in their biological context and measurement format, they do not establish a comparative selectivity profile. Moreover, SIRT4, SIRT6, and SIRT7 were evaluated computationally but were not experimentally assessed in the present study. Direct and matched biochemical, biophysical, and cellular approaches will therefore be required to determine whether the predicted binding modes correspond to functional engagement of individual sirtuin isoforms.

Several additional limitations should be considered when interpreting the structural predictions. The SIRT4 and SIRT7 docking analyses were based on homology models and should therefore be regarded as hypothesis-generating. Although model-quality assessment supported their overall architecture, sensitivity to local pocket geometry, side-chain conformations, and predicted water positions may influence the resulting ligand poses and residue-level contact assignments, particularly for SIRT7. Validation using experimentally determined structures or complementary biochemical and biophysical approaches will therefore be important. Moreover, achieving isoform selectivity among sirtuins remains challenging because differences in loop conformation, pocket architecture, non-conserved local residues, and protein dynamics can influence ligand recognition despite conservation of the catalytic core. Future studies should prioritize experimental determination of BZD9L1-bound SIRT complexes using X-ray crystallography or other appropriate structural approaches. Integration of molecular dynamics simulations with rigorous free-energy calculations, including MM-PBSA or free-energy perturbation, may provide a more comprehensive assessment of protein flexibility, solvent effects, and ligand-induced conformational changes.

Finally, the predicted occupation of the ADP-ribose-binding region raises the possibility that BZD9L1 may compete with NAD^+^ or related cofactors. However, the present docking simulation study does not establish a competitive inhibitory mechanism. Future pharmacological studies should therefore evaluate the influence of NAD^+^ concentration on BZD9L1 activity and assess its interactions with other NAD^+^-dependent enzymes.

## Figures and Tables

**Table 1:** Quality assessment of SIRT4 and SIRT7 homology models.

Score/Model	SIRT4	SIRT7
^1^ Ramachandran plot region (%): Favored	98.6	94.1
Allowed	1.1	3.7
Outlier	0.4	2.2
^2^ Overall model quality z-score	−6.90	−6.38
^3^ Global QMEAN z-score	−0.27	−2.67
^4^ MolProbity score	1.17	1.96
^4^ Clash score (>0.45 Å non-H-bond)	3.39	8.60
^4^ Cβ deviation (>0.45 Å; number of residues)	0	2
^5^ Cα RMSD (Å)	0.76	1.02

^1^ RAMPAGE, ^2^ ProSA−web, ^3^ SWISS-MODEL Structure Assessment Tools, ^4^ MolProbity, ^5^ PyMOL.

## Data Availability

The original contributions presented in this study are included in the article/Supplementary Materials. Further inquiries can be directed to the corresponding authors.

## Supplementary Materials

The following supporting information can be downloaded at: https://www.mdpi.com/article/10.3390/ijms27156840/s1.
