# Supplementary material for: Comparative Structure-Based Analysis of Predicted BZD9L1 Binding Modes Across Human Sirtuins
Source: Int J Mol Sci. 2026 Jul 30;27(15):6840. doi: 10.3390/ijms27156840 (PMC13467308; doi:10.3390/ijms27156840)
Supplement: Supplementary file 1 [file ijms-27-06840-s001.zip › Supplementary Figure S9.pdf]

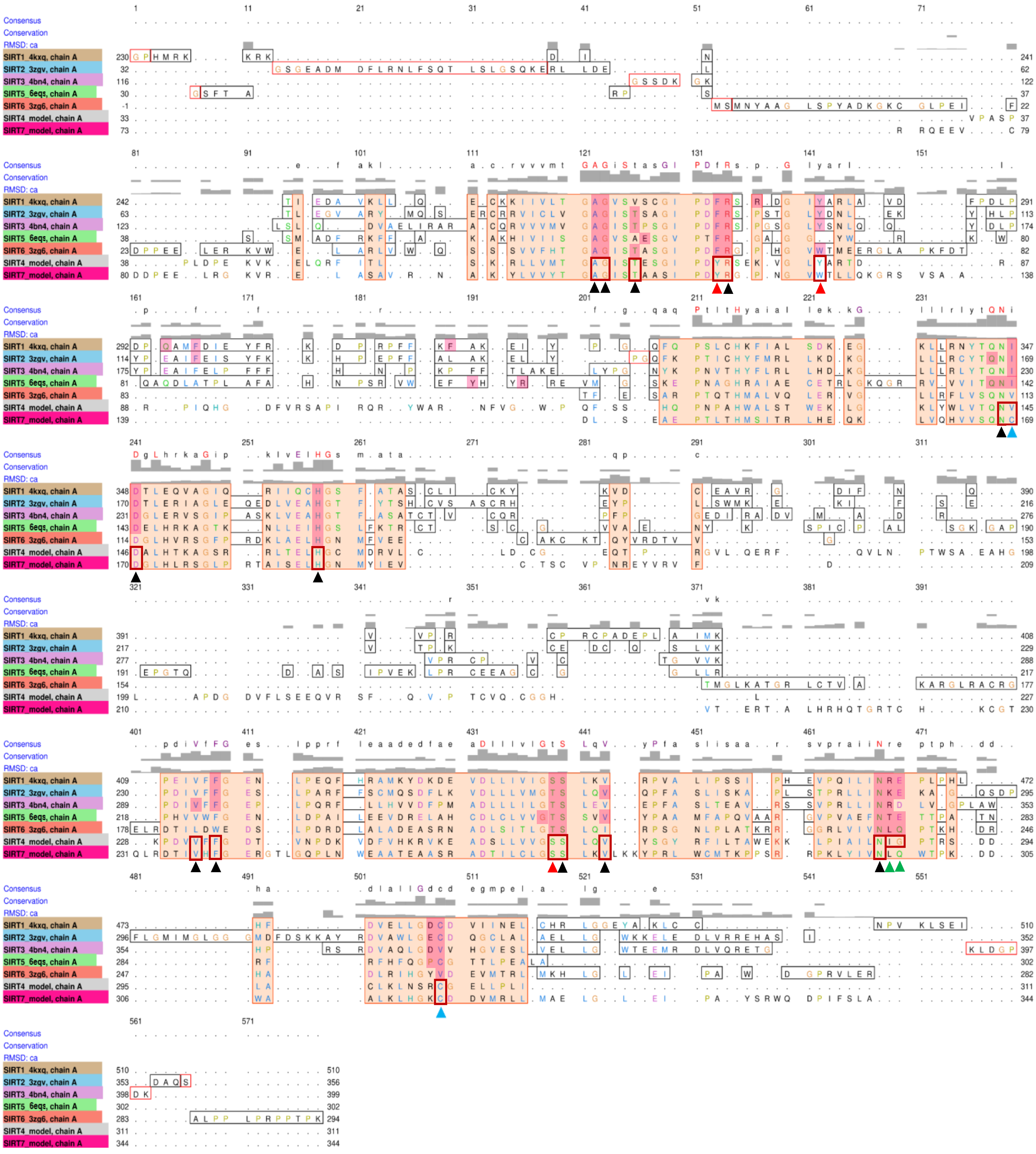

Supplementary Figure S9. Multiple sequence alignment of the cofactor-binding regions of SIRT1–7 used to identify conserved residues and candidate flexible side chains for molecular docking. X-ray diffraction crystal structures (SIRT1, 2, 3, 5 and 6) and homology models (SIRT4 and 7) were structurally superimposed using MatchMaker and then sequence-aligned with Match->Align in UCSF Chimera. The region show in light salmon colour represents conserved amino acids, the pink shaded regions highlight residues interacting with ADPR in the respective crystal structures. Dark red boxes highlight residues predicted to be predicted to contribute to ligand recognition based on structural alignment of the cofactor-binding regions based on alignment of the active site backbone. Colour coding of residues is based on Clustal X: Black arrows for identical, red arrows for conserved, blue arrows for semi-conserved, and green arrows for non-conserved res-idues in the homology models compared to the protein crystal structures.
