# Supplementary material for: Comparative Structure-Based Analysis of Predicted BZD9L1 Binding Modes Across Human Sirtuins
Source: Int J Mol Sci. 2026 Jul 30;27(15):6840. doi: 10.3390/ijms27156840 (PMC13467308; doi:10.3390/ijms27156840)
Supplement: Supplementary file 1 [file ijms-27-06840-s001.zip › Table S1.pdf]

Supplementary Table S1. AutoDock Vina-predicted binding energies for the selected BZD9L1 docking poses across SIRT1–7.

SIRT (PDB ID)	Presence of active site water molecules, docking pose rank and free energies of binding of docked complexes [$\Delta G_{binding}$ (kcal/mol)]			
SIRT1 (4KXQ)	HOH-712 and HOH-835	HOH-712	HOH-835	No water
	R4; -10.60	R1; -11.20	R5; -9.90	R2; -11.20§
SIRT2 (3ZGV)	HOH-2025 and HOH-2105	HOH-2025	HOH-2105	No water
	R1; -11.10	R9; -10.20*	R1; -11.10	R1; -10.90§
SIRT3 (4BN4)	HOH-2024 and HOH-2198	HOH-2024	HOH-2198	No water
	R1; -11.20	R1; -11.10*	R1; -11.20	R1; -11.10§
SIRT4	HOH-575 and HOH-657	HOH-575	HOH-657	No water
	R3; -10.50§	R9; -9.90	R6; -10.40	-
SIRT5 (6EQS)	HOH-573 and HOH-633	HOH-573	HOH-633	No water
	R5; -9.60	-	R5; -9.60§	-
SIRT6 (3ZG6)	HOH-2035 and HOH-2041	HOH-2035	HOH-2041	No water
	R2; -10.40	R2; -10.60*	R2; -10.40	R2; -10.60§
SIRT7	HOH-369 and HOH-457	HOH-369	HOH-457	No water
	R4; -9.70	R7; -10.20	R5; -9.60	R9; -10.20§

‘*’ Water molecule located outside the BZD9L1 binding site and did not affect binding.
‘-’ No favourable docking conformation was attained.
‘§’ Selected docked pose.

AutoDock Vina-predicted binding energies are empirical docking scores expressed in kcal mol^{-1} and were used for pose selection and qualitative comparison rather than as experimentally determined thermodynamic binding free energies.
