# Supplementary material for: Comparative Structure-Based Analysis of Predicted BZD9L1 Binding Modes Across Human Sirtuins
Source: Int J Mol Sci. 2026 Jul 30;27(15):6840. doi: 10.3390/ijms27156840 (PMC13467308; doi:10.3390/ijms27156840)
Supplement: Supplementary file 1 [file ijms-27-06840-s001.zip › Table S2.pdf]

Supplementary Table S2. Predicted residue-level contacts identified in the selected BZD9L1 docking poses across SIRT1–7.

Type of interaction	BZD9L1-SIRT complex/number of predicted contacts or proximal residues [residue number (interaction distance in Å)]						
	SIRT1	SIRT2	SIRT3	SIRT4	SIRT5	SIRT6	SIRT7
Hydrogen bond	**3** [Y280 (2.69)], [Q294 (2.56)], [H363 (2.72)]	**3** [R97 (2.26)], [Y104 (2.64)], [H187 (2.40)]	**4** [R158 (2.28)], [R158 (2.68)], [Y165 (2.75)], [H248 (2.97)]	**2** [R75 (2.47)], [HOH-657 (2.59)]	**1** [Q140 (2.72)]	**3** [R63 (2.61)], [H131 (1.96)], [S214 (1.90)]	**1** [R279 (2.16)]
Carbon hydrogen / pi contact	**1** [V412 (2.55)]	**1** [S98 (2.46)]	**1** [D156 (2.69)]	**2** [D73 (1.93)], [S262 (2.74)]	**1** [V221 (2.43)]	**-**	**1** [Q299 (2.43)]
Pi-pi stacking	**3** [F273 (3.81)], [F273 (4.08)], [H363 (4.97)]	**2** [F96 (4.71)], [F96 (5.21)]	**2** [F157 (3.77)], [F157 (4.43)]	**2** [Y74 (4.86)], [Y74 (5.17)]	**2** [F70 (5.09)], [F70 (5.20)]	**-**	**1** [Y119 (4.76)]
Hydrophobic contact	**4** [A262 (4.22)], [F273 (4.86)], [F297 (3.69)], [I411 (5.24)]	**8** [A85 (4.90)], [A85 (4.49)], [F96 (4.83)], [R97 (5.47)], [F119 (4.08)], [H187 (4.92)], [V266 (5.08)], [K287 (5.30)]	**8** [A146 (4.37)], [A146 (4.58)], [A146 (5.32)], [R158 (5.19)], [F180 (3.75)], [I230 (4.36)], [H248 (4.98)], [R345 (5.05)]	**7** [P34 (5.41)], [A63 (4.65)], [A63 (4.66)], [R75 (5.35)], [Y74 (4.82)], [F234 (4.90)], [V265 (5.00)]	**7** [A59 (4.40)], [R71 (5.40)], [R71 (5.43)], [F223 (3.70)], [V254 (3.95)], [V254 (4.69)], [V254 (5.34)]	**6** [A51 (4.72)], [A51 (5.36)], [H131 (4.25)], [I217 (3.76)], [I217 (5.47)], [L239 (4.69)]	**9** [A108 (5.18)], [A108 (5.33)], [R120 (4.49)], [R120 (5.19)], [R120 (5.42)], [W126 (4.89)], [K272 (3.80)], [L274 (4.39)], [L298 (4.88)]
Pi-cation or charged-residue proximity (electrostatic)	**-**	**-**	**-**	**1** [D73 (4.92)]	**-**	**2** [D61 (3.65)], [R63 (3.60)]	**2** [D118 (3.77)], [R279 (3.97)]
Proximal residues without an assigned interaction	**9** [G263], [D272], [R274], [Q345], [G440], [S441], [S442], [N465], [E467]	**9** [G86], [D95], [Q167], [F235], [G261], [T262], [S263], [N286], [E288]	**8** [G147], [D159], [Q228], [G319], [T320], [S321], [N344], [D346]	**10** [G64], [S76], [Q143], [H161], [V232], [G260], [S261], [S262], [N286], [G288], [HOH-575]	**8** [G60], [T69], [H158], [G249], [T250], [S251], [N275], [T276], [HOH-633]	**9** [E20], [G52], [F62], [G64], [P65], [Q111], [G212], [N238], [Q240]	**7** [E77], [G109], [G121], [G268], [S269], [S270], [N297]

Contacts were assigned from the selected docking poses using the geometric criteria described in the Materials and Methods and do not represent experimentally determined protein–ligand interactions. Carbon hydrogen/pi contacts were classified as weak noncovalent contacts and were not considered conventional hydrogen bonds. Pi-pi stacking denotes a predicted noncovalent interaction between aromatic ring systems based on their relative distance and orientation in the selected docking pose. Charged-residue proximities are reported as geometric observations in the selected docking poses and do not imply energetically favourable π–anion interactions. Proximal residues without an assigned interaction were located near BZD9L1 in the selected docking poses but did not satisfy the geometric criteria used to assign a specific molecular contact.
