# Supplementary material for: Comparative Structure-Based Analysis of Predicted BZD9L1 Binding Modes Across Human Sirtuins
Source: Int J Mol Sci. 2026 Jul 30;27(15):6840. doi: 10.3390/ijms27156840 (PMC13467308; doi:10.3390/ijms27156840)
Supplement: Supplementary file 1 [file ijms-27-06840-s001.zip › Table S3.pdf]

Supplementary Table S3a. AutoDock Vina grid-box parameters used for ADPR redocking and BZD9L1 docking across SIRT1-7. Grid centres and dimensions are reported in Å. A grid spacing of 0.375 Å was used.

Isoform	BZD9L1 grid centre, X/Y/Z	BZD9L1 grid size, X/Y/Z	ADPR grid centre, X/Y/Z	ADPR grid size, X/Y/Z
SIRT1	29.777, -17.225, 10.178	24.750, 24.000, 25.500	29.777, -17.032, 10.178	24.750, 25.125, 25.500
SIRT2	8.184, 28.002, -2.215	25.000, 25.000, 25.000	8.184, 28.002, -2.215	25.000, 25.000, 25.000
SIRT3	12.684, 4.917, 2.403	20.625, 18.000, 21.000	11.342, 2.746, 2.791	24.375, 24.000, 21.000
SIRT4	28.553, 9.037, 48.420	23.250, 25.500, 22.500	28.553, 9.037, 48.420	23.250, 25.500, 22.500
SIRT5	9.877, 15.657, -10.078	23.250, 35.500, 25.500	9.877, 15.657, -10.078	23.250, 35.500, 25.500
SIRT6	31.871, -26.950, 22.078	19.500, 22.500, 18.750	31.871, -26.950, 22.078	19.500, 22.500, 18.750
SIRT7	10.682, -8.829, 22.634	24.750, 19.500, 21.750	9.568, -8.829, 22.634	29.250, 19.500, 21.750

Supplementary Table S3b. Isoform-specific residues treated as flexible during ADPR redocking and BZD9L1 docking. Residues not listed were retained as part of the rigid receptor. The flexible-residue sets were ligand-specific and therefore differed between ADPR and BZD9L1 calculations.

Isoform	Ligand	Flexible residues
SIRT1	BZD9L1	A262, G263, D272, F273, R274, S275, Y280, Q294, F297, H363, F414, S441, S442
SIRT2	BZD9L1	A85, F96, R97, Y104, F119, H187, F235, T262, S263, V266
SIRT3	BZD9L1	A146, G147, T150, F157, R158, Y165, I230, V292, F294, T320, S321, V324, R345
SIRT4	BZD9L1	A63, Y74, R75, Y82, N144, V145, V232, F234, S261, S262, V265
SIRT5	BZD9L1	A59, F70, Y102, R105, Q140, N141, I142, S251, V254, T276
SIRT6	BZD9L1	A51, T55, F62, R63, T213, S214, N238, L239, Q240
SIRT7	BZD9L1	A108, Y119, R120, W126, N168, C169, F239, S269, S270, V273
SIRT1	ADPR	A262, G263, F273, R274, R276, Y280, Q294, F297, H363, F414, S441, S442, E467, D481, C482
SIRT2	ADPR	A85, F96, R97, Y104, F119, H187, F235, T262, S263, V266, N286, E288
SIRT3	ADPR	A146, G147, T150, F157, R158, Y165, I230, V292, F294, T320, R345, D365, V366
SIRT4	ADPR	A63, Y74, R75, Y82, N144, V145, D146, V232, F234, S261, V265, I287
SIRT5	ADPR	A59, F70, Y102, R105, Q140, N141, I142, S251, T276, P292, C293
SIRT6	ADPR	A51, T55, F62, R63, T213, S214, N238, L239, Q240, V256
SIRT7	ADPR	A108, Y119, R120, W126, C169, D170, F239, S269, V273, L298

Flexible-residue sets were selected separately for ADPR and BZD9L1 according to their positions within the corresponding docking grids, the availability of rotatable side-chain torsions, intramolecular interactions, and proximity to the ligand-binding region. Inclusion in the flexible set indicates that side-chain torsional adjustment was permitted during docking.
