# Supplementary material for: Comparative Structure-Based Analysis of Predicted BZD9L1 Binding Modes Across Human Sirtuins
Source: Int J Mol Sci. 2026 Jul 30;27(15):6840. doi: 10.3390/ijms27156840 (PMC13467308; doi:10.3390/ijms27156840)
Supplement: Supplementary file 1 [file ijms-27-06840-s001.zip › Supplementary Figure S1.pdf]

SIRT4 model

(a)

```
CLUSTAL O(1.2.4) multiple sequence alignment


sp|Q9Y6E7|SIR4_HUMAN      MKMSFALTFRSAKGRWIANPSQPCSKASIGLFVPASPPLDPEKVKELQRFITLSKRLLVM  60
pdb|5OJ7|A                ------------------------------HMVPACPPPNPHQVEQLQDFVSQSQRLFVM  30
                                                         :***.** :*.:*::** *:: *:**:**

sp|Q9Y6E7|SIR4_HUMAN      TGAGISTESGIPDYRSEKVGLYARTDRRPIQHGDFVRSAPIRQRYWARNFVGWPQFSSHQ  120
pdb|5OJ7|A                TGAGISTESGIPDYRSEGVGLYSRTERRPIQHSEFVQSQAARRRYWARNFVGWPSFSSHE  90
                          ***************** ****:**:******.:**:*   *:***********.****:

sp|Q9Y6E7|SIR4_HUMAN      PNPAHWALSTWEKLGKLYWLVTQNVDALHTKAGSRRLTELHGCMDRVLCLDCGEQTPRGV  180
pdb|5OJ7|A                PNSAHVNLCKWERAGRLHWLVTQNVDALHTKAGQCRLSELHGCTHRVICLGCQTVTKRSE  150
                          ** **  *..**: *:*:***************. **:***** .**:**.*   * *.

sp|Q9Y6E7|SIR4_HUMAN      LQERFQVLNPTWSAEAHGLAPDGDVFLSEEQVRSFQVPTCVQCGGHLKPDVVFFGDTVNP  240
pdb|5OJ7|A                LQERFLNLNPSWNEQAHGLAPDGDVFLTDEQVSDFQVPACTKCGGILKPQVTFFGDTVNR  210
                          *****  ***:*. :************::*** .****:*.:*** ***:*.*******

sp|Q9Y6E7|SIR4_HUMAN      DKVDFVHKRVKEADSLLVVGSSLQVYSGYRFILTAWEKKLPIAILNIGPTRSDDLACLKL  300
pdb|5OJ7|A                GFVFSIYEQMKQADAMLIVGSSLQVYSGYRFALNAKELHLPIAILNIGPTRADHLAKVKV  270
                          . *  :::::*:**::*:************* *.* * :************:*.** :*:

sp|Q9Y6E7|SIR4_HUMAN      NSRCGELLPLIDPC--      314
pdb|5OJ7|A                SARCGDVLPHILLQDQ      286
                          .:***::** *
```

(c)

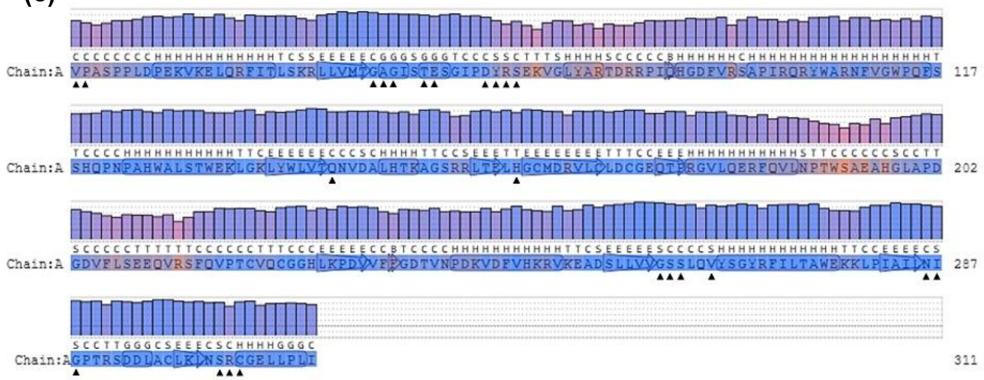

SIRT7 model

(b)

```
CLUSTAL O(1.2.4) multiple sequence alignment


sp|Q9NRC8|SIR7_HUMAN      MAAGGLSRSERKAAERVRRLREEQQRERLRQVSRILRKAAAERSAEEGRLLAESADLVTE  60
3PKI_A                    -------------------------------------------------------GSVNY  5
                                                                                 . *.

sp|Q9NRC8|SIR7_HUMAN      LQGRSRRREGLKRRQEEVCDDPEELRGKVRELASAVRNAKYLVVYTGAGISTAASIPDYR  120
3PKI_A                    AAGLSPYADKGKCGLPEIFDPPEELERKVWELARLVWQSSSVVFHTGAGISTASGIPDFR  65
                            * *   :  *    *: * ****. ** ***  * ::. :*.:********:.***:*

sp|Q9NRC8|SIR7_HUMAN      GPNGVWTLLQKGRSVSA-ADLSEAEPTLTHMSITRLHEQKLVQHVVSQNCDGLHLRSGLP  179
3PKI_A                    GPHGVWTMEERGLAPKFDTTFESARPTQTHMALVQLERVGLLRFLVSQNVDGLHVRSGFP  125
                          **:****: ::* : .  : :..*.** ***::.:*..  *::.:**** ****:***:*

sp|Q9NRC8|SIR7_HUMAN      RTAISELHGNMYIEVCTSCVPNREYVRVFD---VTERTALHRHQTGRTCHKCGTQLRDTI  236
3PKI_A                    RDKLAELHGNMFVEECAKCKTQYVRDTVVGTMGLKATGRLCTVAKARGLRACRGELRDTI  185
                          *  ::******::* *:.*  :     *..   :.    *    ..*  : *  :*****

sp|Q9NRC8|SIR7_HUMAN      VHFGERGTLGQPLNWEAATEAASRADTILCLGSSLKVLKKYPRLWCMTKPPSRRPKLYIV  296
3PKI_A                    LDWEDSLPDR---DLALADEASRNADLSITLGTSLQIRPSGN---LPLATKRRGGRLVIV  239
                          :.: :        :   * **: .**  :  **:**::  .            *  :* **

sp|Q9NRC8|SIR7_HUMAN      NLQWTPKDDWAALKLHGKCDDVMRLLMAELGLEIPAYSRWQDPIFSL---A---TPLRAG  350
3PKI_A                    NLQPTKHDRHADLRIHGYVDEVMTRLMEHLGLEIPAWDGPRVLERALPPLPRPPTPKLEP  299
                          *** * :*  * *::**  *:**  ** .*******:.  :    :*       **

sp|Q9NRC8|SIR7_HUMAN      EEGSH----------SRKSLCRSREEAPPG--DRGAPLSSAPILGGWFGRGCTKRTKRKK  398
3PKI_A                    KEESPTRINGSIPAGPKQEPCAQHNGSEPASPKRERPTSPAPHR-------PPKRVKAKA  352
                          :* *            ::. * .:: : *.  .*  * * **           **.* *

sp|Q9NRC8|SIR7_HUMAN      VT-   400
3PKI_A                    VPS   355
                          *
```

(d)

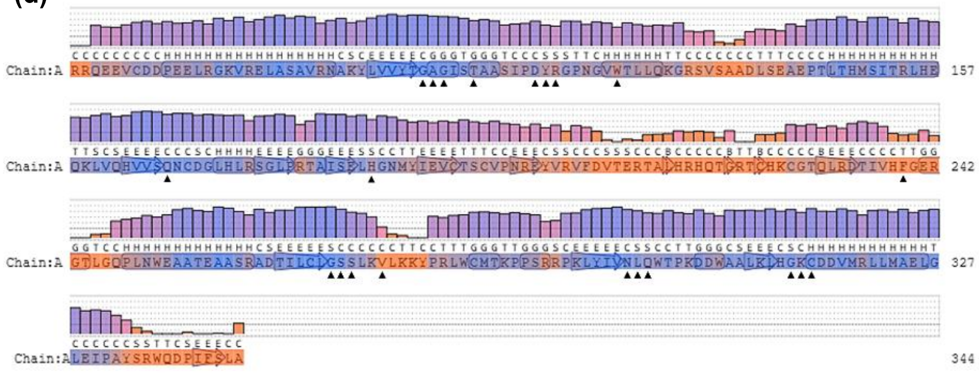

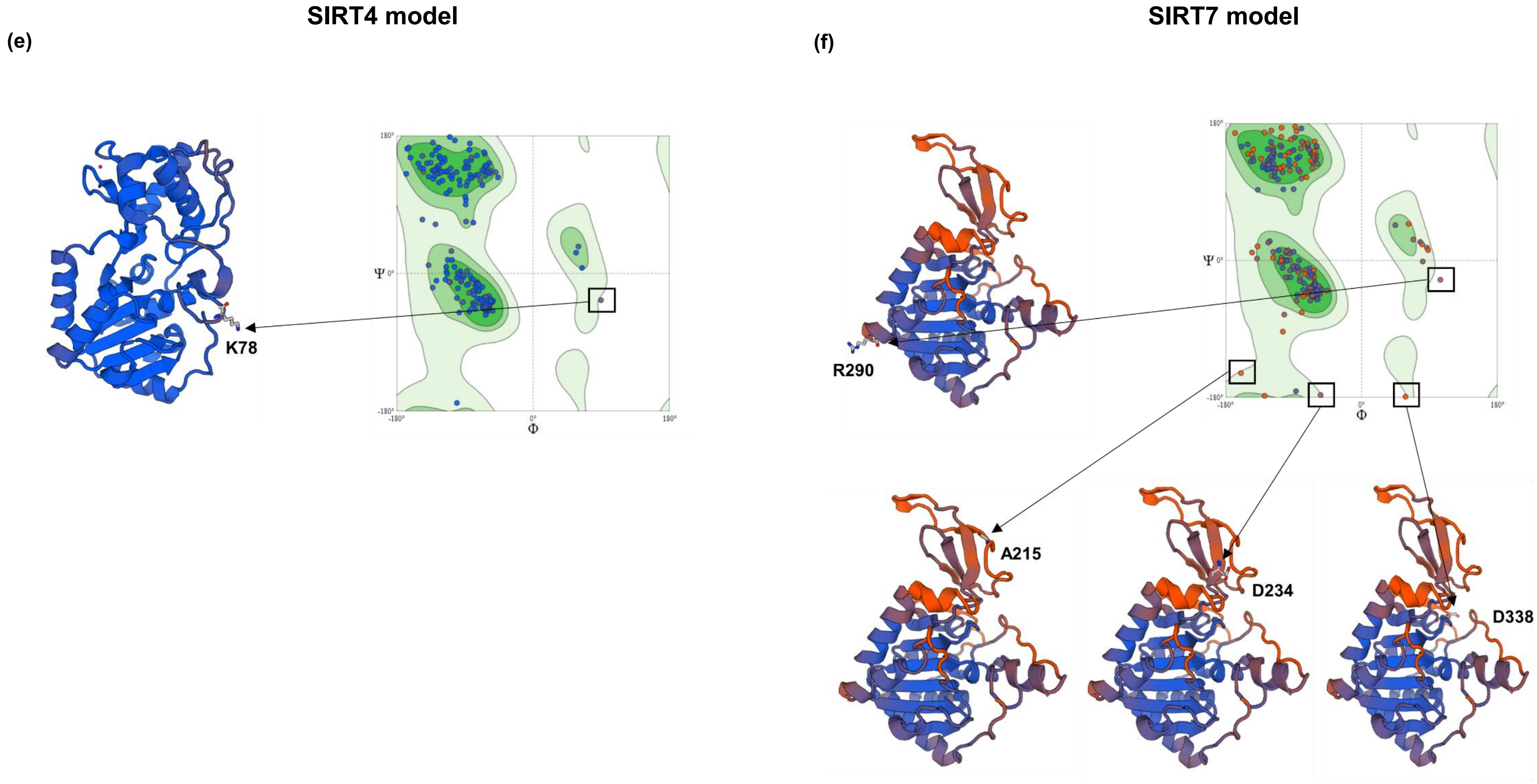

Supplementary Figure S1. Sequence, structural-quality, and stereochemical assessment of the SIRT4 and SIRT7 homology models and comparison with AlphaFold Database predictions. (a) Human SIRT4 (UniProt ID: Q9Y6E7) and **(b)** SIRT7 (UniProtID: Q9NRC8) proteins were sequence aligned to respective template structures (PDB ID: 5OJ7 and 3PKI) using Clustal Omega server. The symbol "*" indicates identical residues, ":" shows conserved substitutions and "." represents semi-conserved substitutions of amino acids. Per-residue QMEAN values, with values <0.6 shown in progressively warmer colors, are presented for the **(c)** SIRT4 and **(d)** SIRT7 models. Black arrows indicate predicted ADPR-binding residues identified through structural superimposition with the respective template structures. **(e,f)** Ramachandran-outlier residues were mapped onto the SIRT4 and SIRT7 homology models, respectively. The predicted ADPR-binding regions are highlighted in orange, and outlier residues are shown in stick representation and labelled by residue number. The SIRT4 outlier K78 and the SIRT7 outliers A215, D234, R290, and D338 were located outside the predicted ADPR-binding regions.

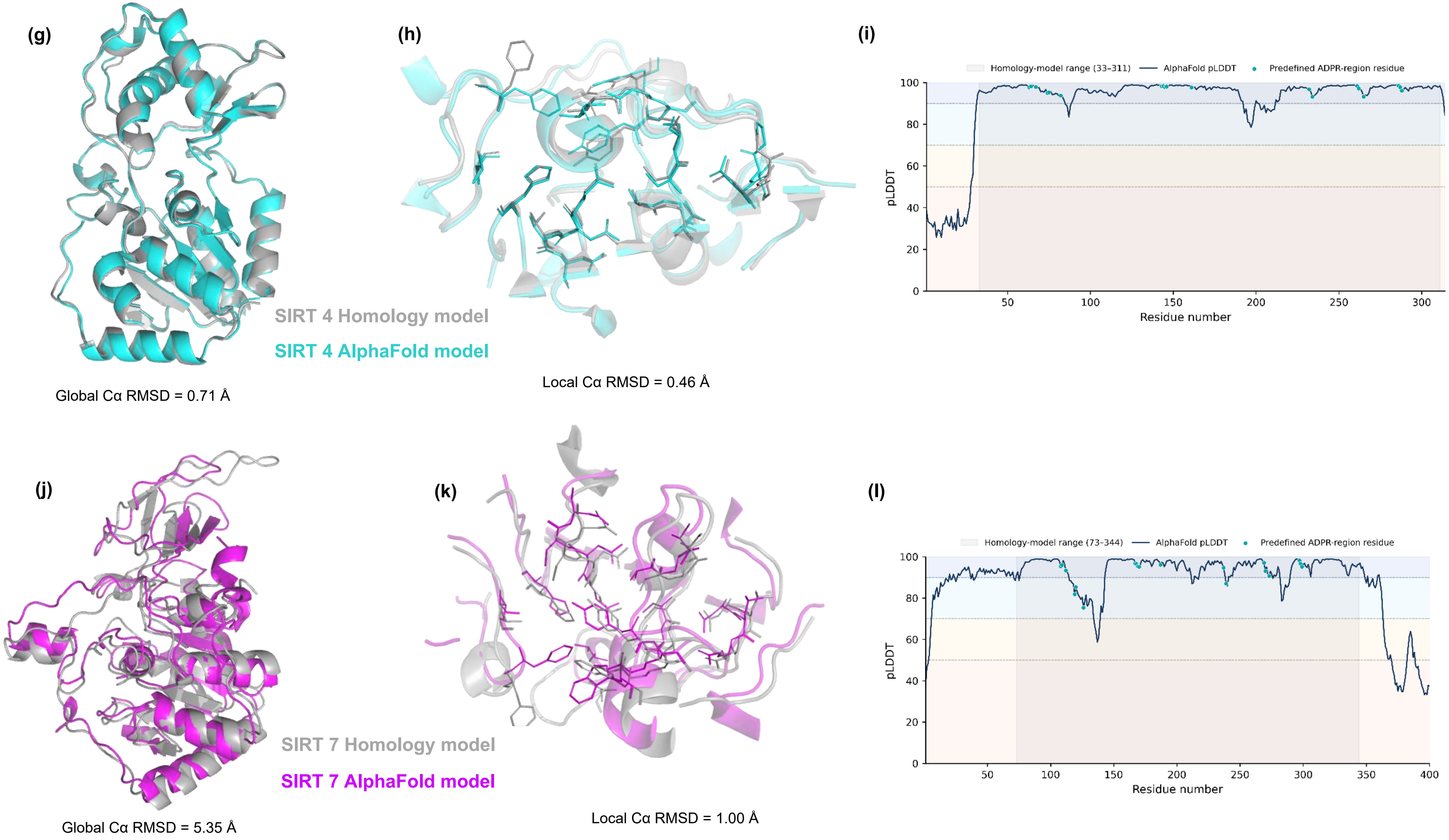

Supplementary Figure S1 (continued). Sequence, structural-quality, and stereochemical assessment of the SIRT4 and SIRT7 homology models and comparison with AlphaFold Database predictions. (g,j) Structural superimposition of the SIRT4 and SIRT7 homology models, respectively, with the corresponding AlphaFold Database predictions. The homology models used for docking are shown in grey, and the AlphaFold models are shown in cyan and magenta. The models were superimposed using corresponding Cα atoms over their common modelled regions. The unpruned global Cα RMSD was 0.71 Å across 279 corresponding residues for SIRT4, residues 33–311, and 5.35 Å across 272 corresponding residues for SIRT7, residues 73–344. **(h,k)** Enlarged views of the predefined SIRT4 and SIRT7 ADPR-binding regions, respectively, following global superimposition. Separate local fitting of the 19 predefined ADPR-region Cα atoms gave RMSD values of 0.46 Å for SIRT4 and 1.00 Å for SIRT7. **(i,l)** Residue-level pLDDT profiles of the SIRT4 and SIRT7 AlphaFold predictions, respectively. Grey shading indicates the region shared with the corresponding homology model, and cyan points indicate the predefined ADPR-region residues. Horizontal dashed lines indicate pLDDT values of 50, 70, and 90. pLDDT values apply only to the AlphaFold predictions and are not quality scores for the homology models. Global Cα RMSD values were calculated without iterative outlier rejection. The enlarged panels show the globally superimposed structures; local fitting was performed separately only to quantify structural agreement within the predefined ADPR-binding regions.
