# Supplementary material for: Comparative Structure-Based Analysis of Predicted BZD9L1 Binding Modes Across Human Sirtuins
Source: Int J Mol Sci. 2026 Jul 30;27(15):6840. doi: 10.3390/ijms27156840 (PMC13467308; doi:10.3390/ijms27156840)
Supplement: Supplementary file 1 [file ijms-27-06840-s001.zip › Supplementary Figure S2.pdf]

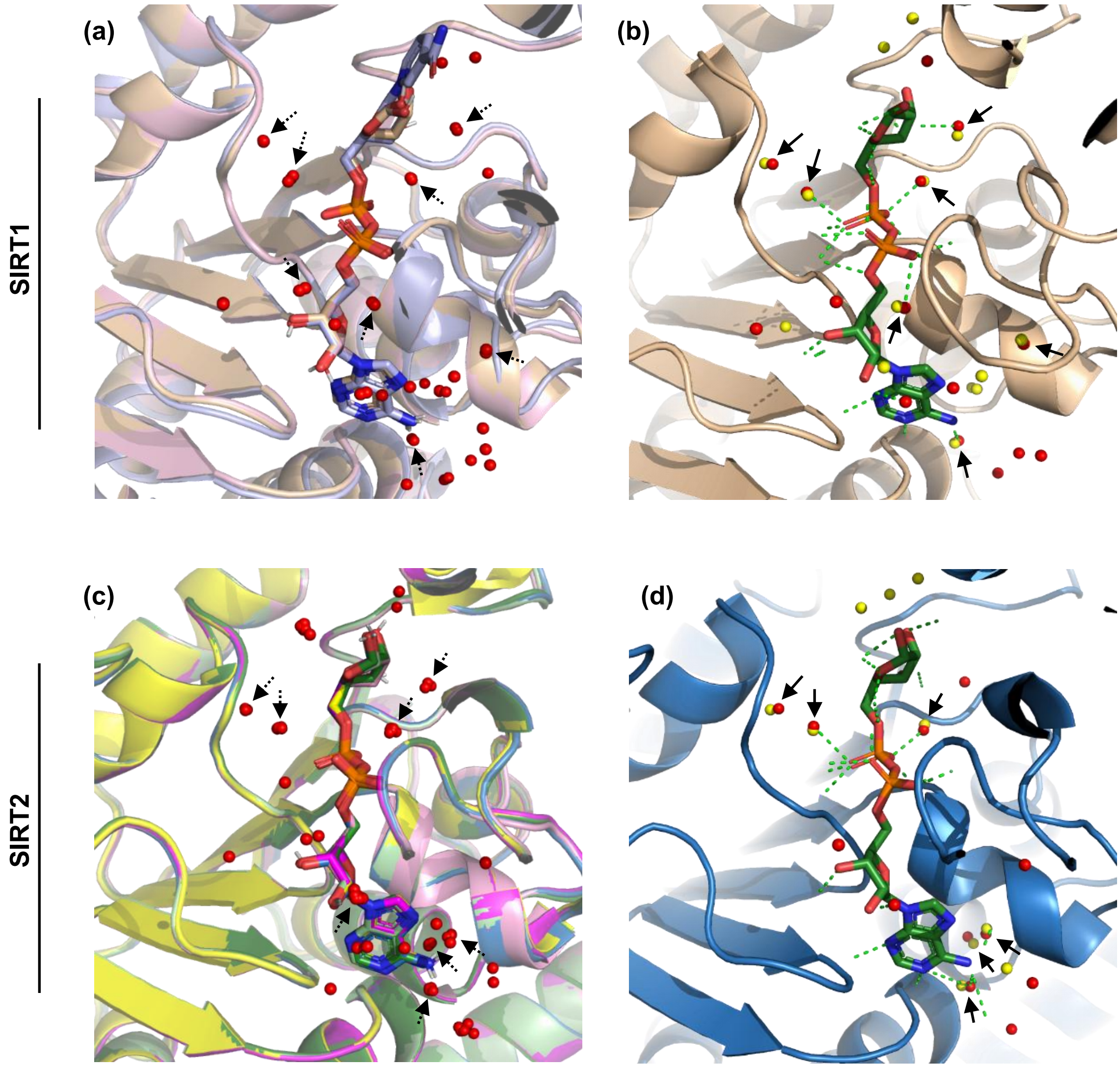

Supplementary Figure S2. Validation of Waterdock 2.0 bridging water algorithm for prediction of locally conserved active site water. (a) Superimposition of SIRT1 crystal structures (PDB ID: 4KXQ, 4I5I and 4IF6) to identify conserved active site crystal water. **(b)** Superimposition of localised water predicted from unsolvated SIRT1 crystal structure (wheat) with native crystal water. **(c)** Superimposition of SIRT2 crystal structures (PDB ID: 3ZGV, 4RMJ, 5D7O, 5D7P, 5D7Q and 5MAR) to identify conserved active site crystal water. **(d)** Superimposition of localised water predicted from unsolvated SIRT2 crystal structure (sky blue) with native crystal water. Predicted water molecules are shown as yellow spheres and crystal water molecules are presented as red spheres. Black arrows indicate direct superimposition of predicted water with crystal water, dotted-lined arrows show conserved crystal water. Prediction of localised water in unsolvated models were performed using Waterdock 2.0 algorithm through AutoDock Vina software. Proteins were presented as cartoon and ligands shown as licorice. Green dotted lines represent hydrogen bond interactions involving ADPR.
