# Supplementary material for: Comparative Structure-Based Analysis of Predicted BZD9L1 Binding Modes Across Human Sirtuins
Source: Int J Mol Sci. 2026 Jul 30;27(15):6840. doi: 10.3390/ijms27156840 (PMC13467308; doi:10.3390/ijms27156840)
Supplement: Supplementary file 1 [file ijms-27-06840-s001.zip › Supplementary Figure S3.pdf]

(a)

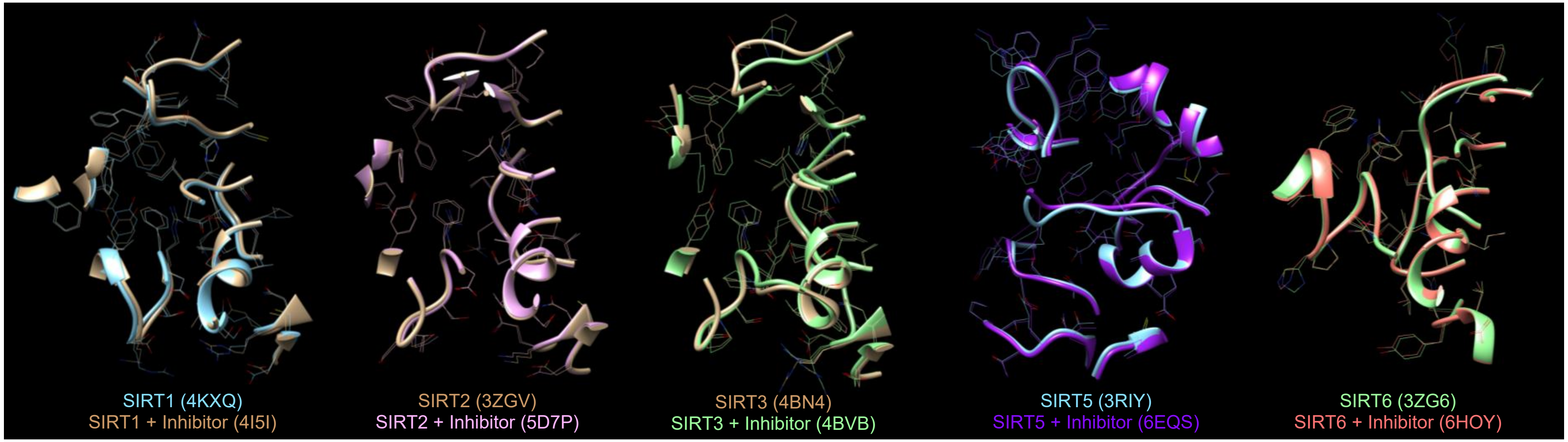

(b)

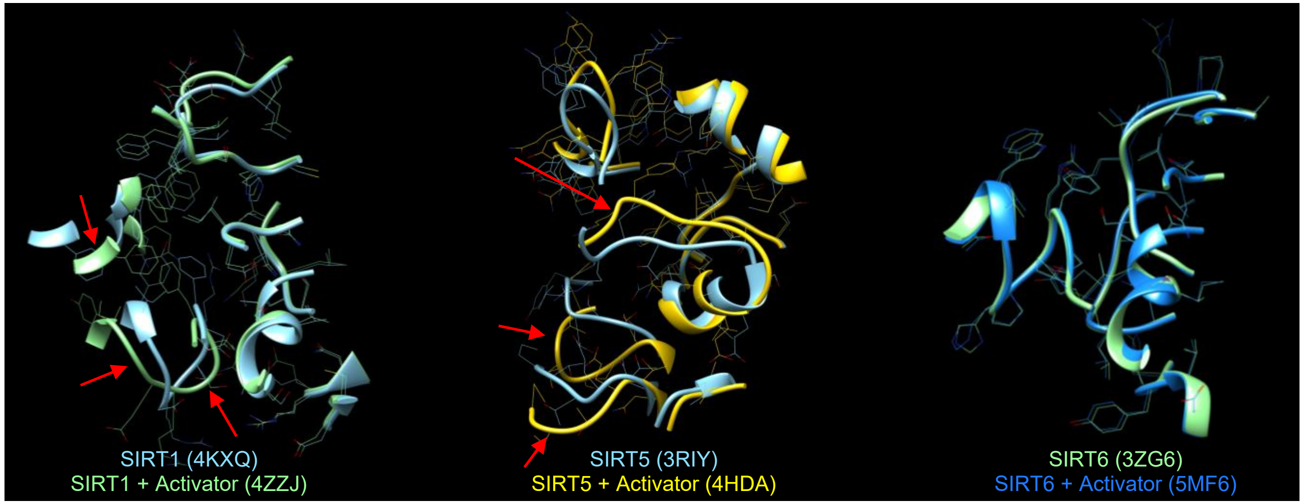

Supplementary Figure S3. Structural comparison of selected SIRT structures with corresponding inhibitor or activator-bound structures. Superimposition of the active-site Cα backbones of the selected reference SIRT crystal structures with corresponding **(a)** inhibitor-bound or **(b)** activator-bound structures. The NAD^+-binding region showed no appreciable backbone deviation in the inhibitor-bound comparisons, whereas the activator-bound structures displayed isoform-dependent conformational differences, including active-site loop displacement in SIRT1 and SIRT5, as indicated by red arrows. Protein Cα backbones are shown as cartoons, and the preselected residues lining or structurally corresponding to the ADPR-binding region are shown as wires. These displayed residues represent the active-site set used for structural comparison and do not exclusively denote side chains treated as flexible during docking. Structural superimposition was performed using UCSF Chimera.
