# Supplementary material for: Comparative Structure-Based Analysis of Predicted BZD9L1 Binding Modes Across Human Sirtuins
Source: Int J Mol Sci. 2026 Jul 30;27(15):6840. doi: 10.3390/ijms27156840 (PMC13467308; doi:10.3390/ijms27156840)
Supplement: Supplementary file 1 [file ijms-27-06840-s001.zip › Supplementary Figure S4.pdf]

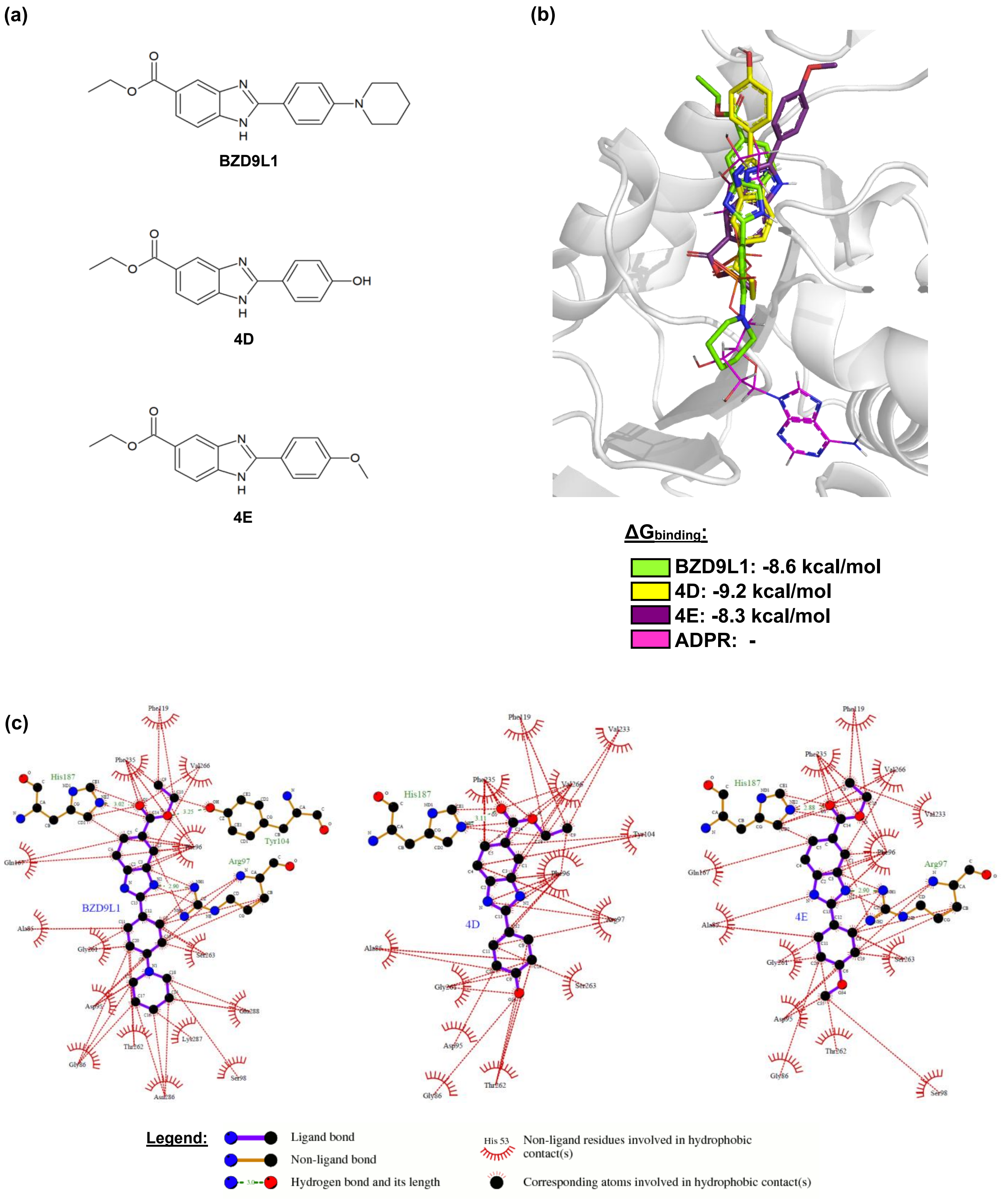

Supplementary Figure S4. Chemical structures and molecular docking of BZD9L1, 4D and 4E compounds. (a) The chemical structures of BZD9L1, 4D and 4E ligands. **(b)** Poses and binding free energies of BZD9L1, 4D, and 4E compounds docked independently into fully rigid SIRT2 protein structure without allowed residue side chain flexibility. Ligands were docked using AutoDock Vina program and superimposed using PyMOL software. **(c)** Interactions involved in docked BZD9L1, 4D, and 4E compounds using AutoDock Vina with allowed SIRT2 residue side chain flexibility were visualized in LigPlot^{+} software.
