# Supplementary material for: Comparative Structure-Based Analysis of Predicted BZD9L1 Binding Modes Across Human Sirtuins
Source: Int J Mol Sci. 2026 Jul 30;27(15):6840. doi: 10.3390/ijms27156840 (PMC13467308; doi:10.3390/ijms27156840)
Supplement: Supplementary file 1 [file ijms-27-06840-s001.zip › Supplementary Figure S5.pdf]

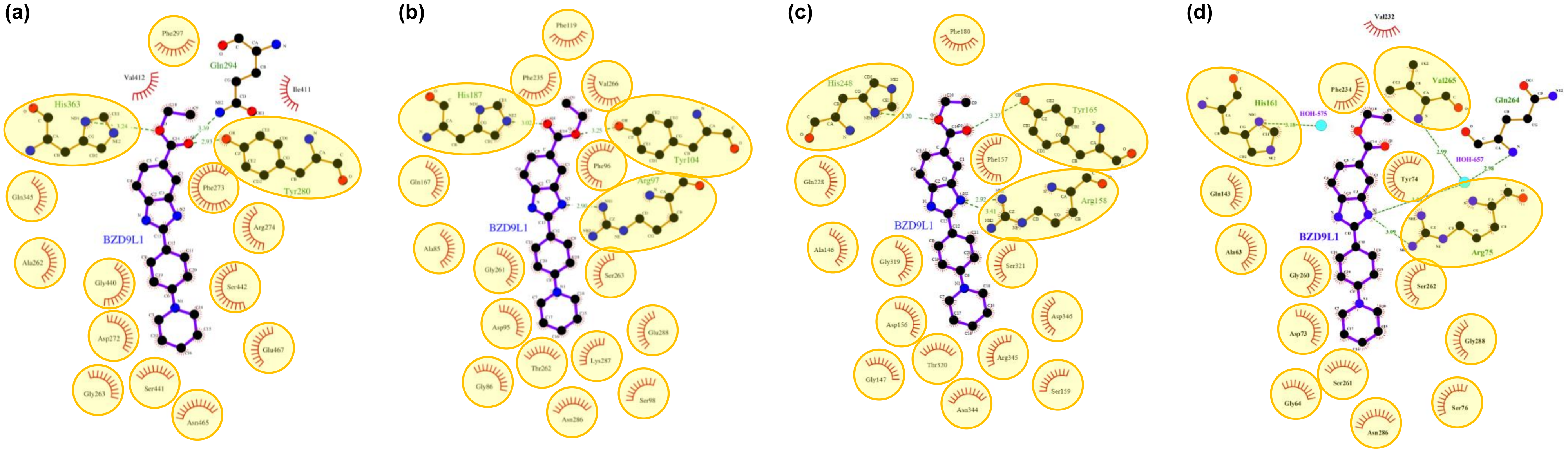

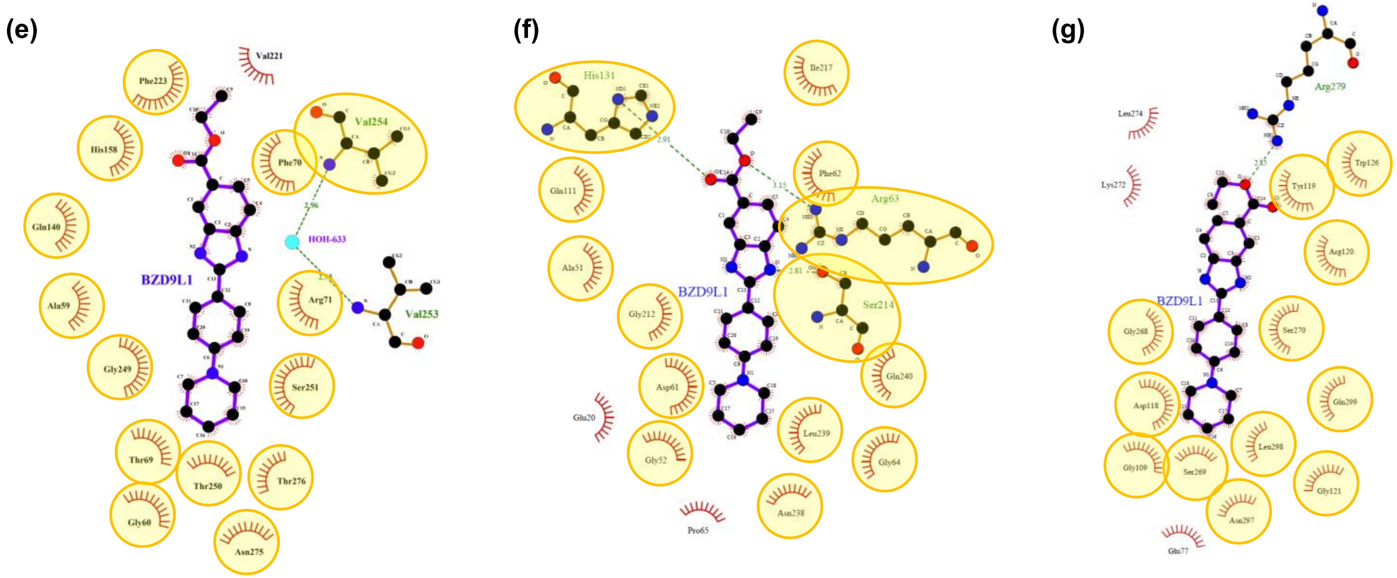

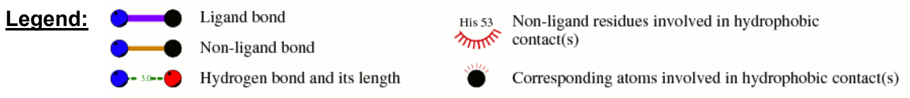

Supplementary Figure S5. The position of BZD9L1-interacting residues in SIRT proteins are highly conserved. Residue side chains interacting with BZD9L1 from docked **(a)** SIRT1, **(b)** SIRT2, **(c)** SIRT3, **(d)** SIRT4, **(e)** SIRT5, **(f)** SIRT6 and **(g)** SIRT7 complexes were assessed using Discovery Studio 4.0 and LigPlot$^+$ software. Yellow shaded circles in each plot indicate amino acid residues with conserved 3D positions. Residues are labelled with three-letter amino acid code followed by residue number. Visualization through LigPlot$^+$ software.
