# Supplementary material for: Comparative Structure-Based Analysis of Predicted BZD9L1 Binding Modes Across Human Sirtuins
Source: Int J Mol Sci. 2026 Jul 30;27(15):6840. doi: 10.3390/ijms27156840 (PMC13467308; doi:10.3390/ijms27156840)
Supplement: Supplementary file 1 [file ijms-27-06840-s001.zip › Supplementary Figure S6.pdf]

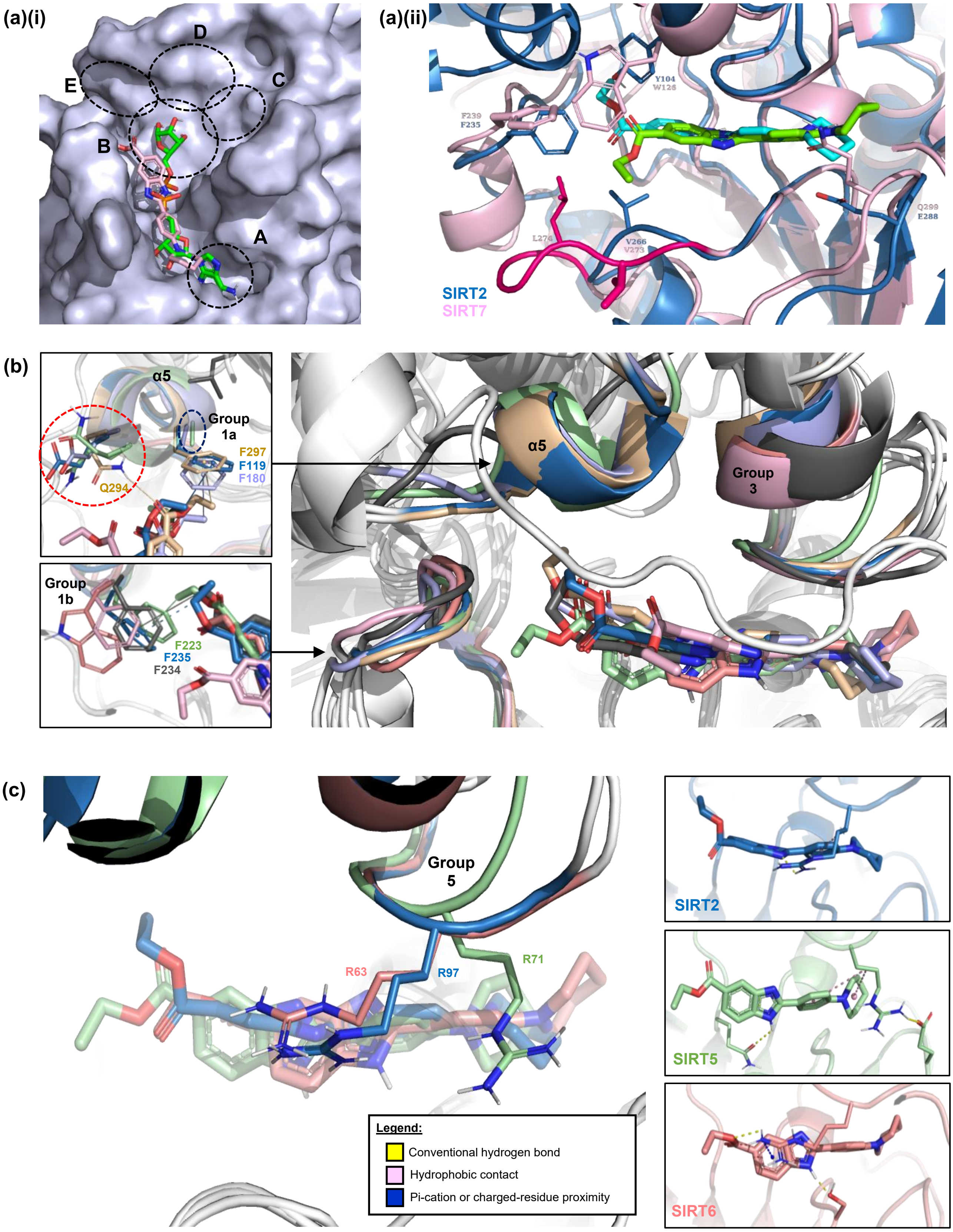


**Supplementary Figure S6. Factors affecting BZD9L1 binding conformation in SIRTs. (a)(i)** BZD9L1 (light pink) did not overlap with docked ADPR (green) at the B-pocket of SIRT7 model due to the **(a)(ii)** presence of an elongated loop (hot pink) that contains three additional residues: L274, K275 and K276. BZD9L1 docked into SIRT7 and SIRT2 are coloured in chartreuse and cyan, respectively. A, B, C, D, and E sites (black circles) are A-pocket, B-pocket, C-pocket, extended C-site, and acetyl-lysine channel, respectively. **(b)** Superimposition of SIRT1 (wheat), SIRT2 (sky blue), SIRT3 (light blue), SIRT4 (grey40), SIRT5 (pale green), SIRT6 (salmon), and SIRT7 (light pink) via the active site backbone chains (Cα). BZD9L1 in all SIRT complexes except for SIRT6 and SIRT7 possessed at least one interaction with residues from either Group 1a, 1b, or an additional group (red circle). In SIRT5, the phenylalanine residue in Group 1a was substituted with an alanine (dark blue circle). Both SIRT6 and SIRT7 structures lacked the α5 helical module. **(c)** The orientation of benzimidazole moiety in docked BZD9L1 was dependent on the position and interaction of conserved arginine within the active site of SIRT complexes. Protein presented as cartoon, BZD9L1 and residue side chains in licorice. Residues are labelled with single-letter amino acid code followed by residue number. Superimposition using UCSF Chimera software and visualization via PyMOL software.
