# Supplementary material for: Comparative Structure-Based Analysis of Predicted BZD9L1 Binding Modes Across Human Sirtuins
Source: Int J Mol Sci. 2026 Jul 30;27(15):6840. doi: 10.3390/ijms27156840 (PMC13467308; doi:10.3390/ijms27156840)
Supplement: Supplementary file 1 [file ijms-27-06840-s001.zip › Supplementary Figure S7.pdf]

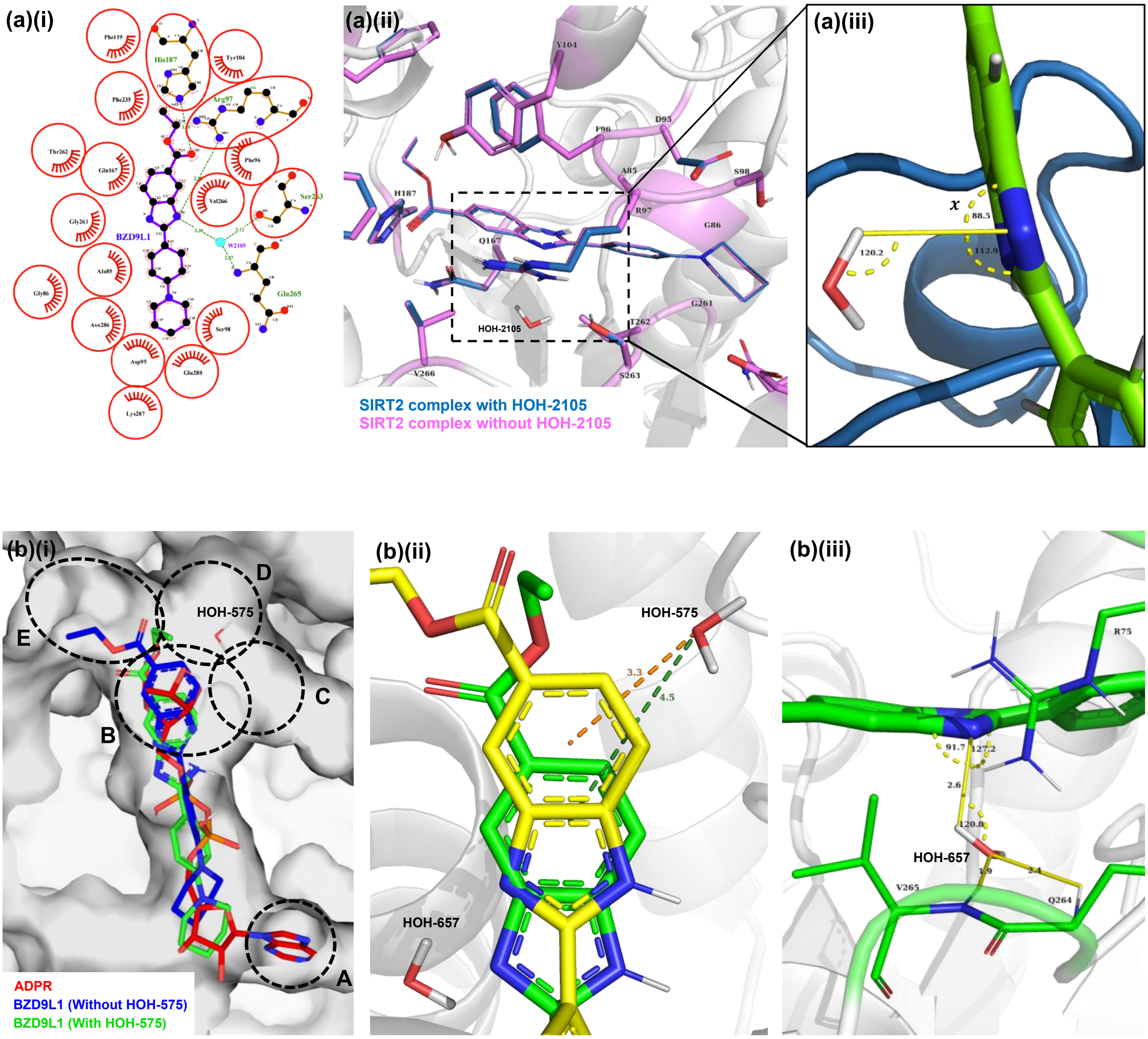

Supplementary Figure S7. Prediction of BZD9L1 interaction with active site water in SIRT2 and SIRT4 proteins. (a)(i) AutoDock Vina predicted the most favorable BZD9L1 binding conformation in SIRT2 protein involved hydrogen bonding with active site water HOH-2105. **(a)(ii)** Spatial conformation of BZD9L1 and interacting residue side chains are identical in complexes with (skyblue) or without (violet) HOH-2105. **(a)(iii)** Analysis of interaction between BZD9L1 and HOH-2105 (magnification from black dotted-lined box) showed ∠H-A-Y angle (represented by $\boldsymbol{x}$) was 88.5° which did not fulfill the pre-set criteria for hydrogen bonding. Direct magnification of the boxed region in a(ii) showing the geometry between BZD9L1 and HOH-2105. The same structural color scheme as in a(ii) was retained without recoloring; any apparent variation in shade results from magnification and three-dimensional rendering effects. Visualisation through LigPlot^{+} and PyMOL software. In (a)(ii), protein presented as cartoon, BZD9L1 as wire, and residues/water as licorice. Structures with water and without water coloured in skyblue and violet, respectively. **(b)(i)** Localization of docked ligands in SIRT4 active site, as divided into A, B, C, D, and E sites (black circles). **(b)(ii)** Superimposition of docked BZD9L1 in SIRT4 structures with both water (green), and only HOH-657 (yellow). Orange and forest dotted-lines are distances between the oxygen atom of HOH-575 and the benzene ring from the benzimidazole moiety of yellow and green-coloured BZD9L1 molecules, respectively. **(b)(iii)** Angles and bond length between BZD9L1 and HOH-575 fulfilled criteria for hydrogen bond formation. The water HOH-575 bridged BZD9L1 to residues Q264 and V265 of SIRT4 protein. Protein presented as cartoon and BZD9L1 as licorice. Residues are labelled with three or single-letter amino acid code followed by residue number. Solid yellow lines are predicted hydrogen bonds. Superimposition, measurements, and visualization through PyMOL software.
