# Supplementary material for: Comparative Structure-Based Analysis of Predicted BZD9L1 Binding Modes Across Human Sirtuins
Source: Int J Mol Sci. 2026 Jul 30;27(15):6840. doi: 10.3390/ijms27156840 (PMC13467308; doi:10.3390/ijms27156840)
Supplement: Supplementary file 1 [file ijms-27-06840-s001.zip › Supplementary Figure S8.pdf]

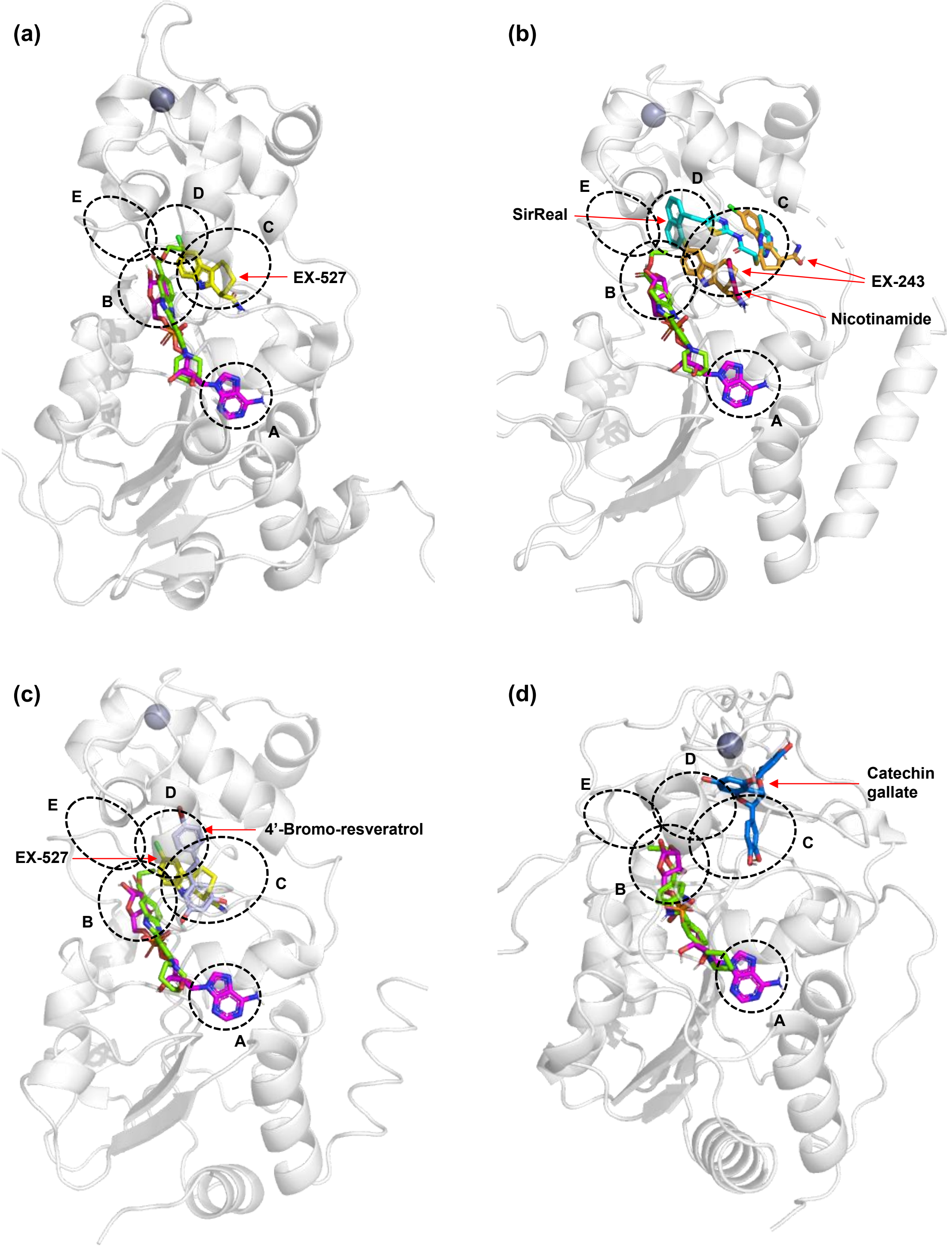

Supplementary Figure S8 Binding conformations of BZD9L1 and other SIRT inhibitors in SIRT proteins. (a) Superimposed conformations of ADPR, BZD9L1, and EX-527 in SIRT1 protein. **(b)** Superimposed conformations of ADPR, BZD9L1, EX-243, Nicotinamide, and SirReal in SIRT2 protein. **(c)** Superimposed conformations of ADPR, BZD9L1, EX-527, and 4-Bromo-resveratrol in SIRT3 protein. **(d)** Superimposed conformations of ADPR, BZD9L1, and Catechin gallate in SIRT6 protein. SIRT protein structures (white) presented as cartoon. A, B, C, D, and E sites (black circles) are A-pocket, B-pocket, C-pocket, extended C-site, and acetyl-lysine channel, respectively. ADPR (magenta), BZD9L1 (chartreuse), EX-527 (yellow), EX-243 (bright orange), SirReal (cyan), Nicotinamide (hot pink), 4'-Bromo-resveratrol (light blue), and Catechin gallate (marine) shown as licorice. Superimposition and visualization via PyMOL software.
